# Supplementary material for: Preferences regarding COVID-19 vaccination among 12,000 adults in China: A cross-sectional discrete choice experiment
Source: PLOS Glob Public Health. 2024 Jul 11;4(7):e0003387. doi: 10.1371/journal.pgph.0003387 (PMC11239003; doi:10.1371/journal.pgph.0003387)
Supplement: S1 Checklist — (DOCX) [file pgph.0003387.s001.docx]

Inclusivity in global research

PLOS’ policy on inclusivity in global research aims to improve transparency in the reporting of research performed outside of researchers’ own country or community and ensures that PLOS publications reporting global research adhere to high standards for research ethics and authorship. Authors of relevant research articles may be asked to complete the questionnaire below, which outlines ethical, cultural, and scientific considerations specific to inclusivity in global research. This questionnaire may be requested when researchers have travelled to a different country to conduct research, if research uses samples collected in another country, research with Indigenous populations or their lands, or if research is on cultural artefacts. Researchers travelling to another country solely to use laboratory equipment will not normally be required to complete the questionnaire. However, the questionnaire can be requested at the journal’s discretion for any submission – if you have been requested to complete this questionnaire by the PLOS journal you submitted to, please do so.

Please complete the questionnaire below and include this as a Supporting Information file with your manuscript. Note that if your paper is accepted for publication, this checklist will be published with your article in the supporting information files. Please ensure that you reference the checklist in the main body of your manuscript. We suggest adding a subsection ‘Inclusivity in global research’ to your Methods section and adding the following sentence: “Additional information regarding the ethical, cultural, and scientific considerations specific to inclusivity in global research is included in the Supporting Information (SX Checklist)”

The questions have been designed to be applicable to a wide range of study types, and there are subsections for both human subjects research and non-human subjects research. If any of the questions are not relevant to your research please mark them as “N/A” as appropriate.

**Ethical considerations, permits and authorship**

*This section is applicable to all research types.*

Provide details as to who granted permissions and/or consent for the study to take place in the Methods section of your manuscript. This should include the names of **all** ethics boards, governmental organizations, community leaders or other bodies that provided approval for the study. If individuals provided approval refer to these people by their role or title but do not list their name(s).

Reported on page number: 11

If there were any deviations from the study protocol after approval was obtained please provide details of these changes in the Methods section of your manuscript.
Did this study involve local collaborators that are residents of the country where the research was conducted or members of the community studied? If you do not have any authors from said communities, please provide an explanation for this below.

Reported on page number: NA. There were no protocol deviations.

Yes. 9 of 17 authors: Fengyun Yu, Qun Wang, Zhong Cao, Wenjin Chen, Xuedi Ma, Chao Wang, Juntao Yang, Lan Xue, Simiao Chen.

Everyone listed as an author should meet PLOS’ criteria for authorship and all individuals who meet these criteria should be included in the author byline, rather than the acknowledgements. For further information please see the journal’s Authorship Policy.

**Human subjects research (e.g. health research, medical research, cross-cultural psychology)**

Did you obtain written informed consent from a representative of the local community or region before the research took place? How did you establish who speaks for the community? Details of written informed consent obtained from study participants should be reported separately in the Methods section of your manuscript.

This study is a online survey distributed by KuRunData, a private online survey company that maintains a database of potential survey participants and administers surveys. Hence, there is no representative of the local community. KuRunData takes advantage of its own platform and partnerships with other websites in recruiting participants, and also encourages registered members to recruit new members through the popular mobile application WeChat Mini. Before filling out the questionnaire, participants provided their informed written consent with signature confirmation. These details have been reported in the Methods section.

How did members of the local community provide input on the aims of the research investigation, its methodology, and its anticipated outcome(s)?

As our research was conducted online and involved a large-scale survey distributed through KuRunData's platform, direct input from members of the local community on the aims, methodology, and anticipated outcomes was not feasible. However, prior to the survey's implementation, we conducted an extensive literature search, consulted with experts in the field, and conducted qualitative interviews to ensure that our research questions were relevant and meaningful. Additionally, we designed the survey instrument to capture a wide range of perspectives and demographic characteristics reflective of the local communities across China, and thus indirectly incorporating input from diverse populations.

When engaging with the local community, how did you ensure that the informed consent documents and other materials could be understood by local stakeholders?

To ensure that our informed consent documents and other materials could be understood by local stakeholders, we employed several strategies. First, we utilized plain language and avoided technical jargon to enhance comprehension. Second, we conducted pilot testing of the consent documents with individuals from diverse backgrounds to identify any potential areas of confusion or misunderstanding. Third, we offered additional support and clarification to participants who expressed difficulty understanding any aspect of the consent documents or study materials.

Will the findings of the research be made available in an understandable format to stakeholders in the community where the study was conducted (e.g. via a presentation, summary report, copies of publications, etc.)? Please provide details of how this will be achieved.

The findings of the research will be disseminated to stakeholders in multiple ways. First, the findings will be published online, and posted on Facebook and X (formerly known as Twitter). The social media posts will be written in plain (non-scientific language) to the general public. Second, the corresponding author Prof. Simiao will present our findings to the Chinesisch-Deutsches Zentrum für Wissenschaftsförderung. Third, we will collaborate with local organizations and institutions to organize workshops or seminars where the findings can be discussed in detail and their implications for the community can be explored. This approach ensures that stakeholders have access to and can engage with the research findings in a meaningful way.
