# Supplementary material for: Preferences regarding COVID-19 vaccination among 12,000 adults in China: A cross-sectional discrete choice experiment
Source: PLOS Glob Public Health. 2024 Jul 11;4(7):e0003387. doi: 10.1371/journal.pgph.0003387 (PMC11239003; doi:10.1371/journal.pgph.0003387)
Supplement: S1 Text — (DOCX) [file pgph.0003387.s002.docx]

## S1 Text: Questionnaire in English and Chinese

**English version**

Questionnaire on Willingness to COVID-19 Vaccine

**Part I: Introduction**

At present the novel coronavirus is still raging all over the world. And the government has adopted a vaccination campaign against COVID-19. We want to know your views and preference regarding vaccine design, specifically the vaccine attributes that are most important to you. This questionnaire consists of five parts: Introduction, Choice experiment, Basic personal information, Health status and vaccination behavior.

We will keep your personal information completely anonymous. Thank you for your support and cooperation!

**Part II: Choice experiment**

Twelve vaccination choice scenarios will now be present to you in the following section. Please state which you would choose in each of these twelve situations (vaccine 1, vaccine 2 or neither).

Please read this information carefully as it changes in every situation. Take your time to read all the information and make your choice accordingly. There is no "right" or "wrong" answer, it's your opinion in each situation that counts.”

**Property description**

Price: total cost of vaccination; free /200 yuan /400 yuan/600 yuan

Risk: the probability of causing some serious side effects (such as thrombus, acute allergic reactions) after vaccination; 0/1 in 1,000,000/1 in 100,000

Protection period: after vaccination, the vaccine will maintain its intended effectiveness for a period of time (the effectiveness may decrease after the protection period is exceeded); 6 months/12 months/life time

Efficacy: Compared with people who have not been vaccinated, the proportion of susceptible people after vaccination reduced by the probability of virus infection; 50%, 70%, 90%

Vaccination method: injection / oral

Frequency of vaccination: total number of doses need to take; 1 time/2 times/3 times

Vaccine origin: imported/domestic

|  | **Block 1** |  |  | **Block 2** |  |  | **Block 3** |  |  |
| --- | --- | --- | --- | --- | --- | --- | --- | --- | --- |
| **Question 1** |  | **vaccine1** | **vaccine2** |  | **vaccine1** | **vaccine2** |  | **vaccine1** | **vaccine2** |
|  | **price** | 0 | 400 | **price** | 600 | 200 | **price** | 0 | 200 |
|  | **risk** | 1/1000000 | 0 | **risk** | 0 | 1/100000 | **risk** | 1/100000 | 1/1000000 |
|  | **duration** | Life long | 6 months | **duration** | 6 months | 12 months | **duration** | 12 months | 12 months |
|  | **efficacy** | 90% | 90% | **efficacy** | 70% | 50% | **efficacy** | 70% | 70% |
|  | **admin** | Oral | Oral | **admin** | Oral | Oral | **admin** | Injection | Injection |
|  | **doses** | 2 doses | 1 dose | **doses** | 2 doses | 2 doses | **doses** | 3 doses | 3 doses |
|  | **origin** | Imported | Domestic | **origin** | Imported | Domestic | **origin** | Domestic | Domestic |
|  | **Choice question:** |  |  | **Choice question:** |  |  | **Choice question:** |  |  |
| **Question 2** |  | **vaccine1** | **vaccine2** |  | **vaccine1** | **vaccine2** |  | **vaccine1** | **vaccine2** |
|  | **price** | 200 | 200 | **price** | 600 | 0 | **price** | 0 | 0 |
|  | **risk** | 1/1000000 | 1/100000 | **risk** | 1/100000 | 0 | **risk** | 1/100000 | 0 |
|  | **duration** | 12 months | life long | **duration** | 12 months | life long | **duration** | Life long | 6 months |
|  | **efficacy** | 70% | 90% | **efficacy** | 50% | 90% | **efficacy** | 90% | 50% |
|  | **admin** | Injection | Injection | **admin** | Injection | Injection | **admin** | Injection | Injection |
|  | **doses** | 3 doses | 1 dose | **doses** | 1 dose | 1 dose | **doses** | 2 doses | 3 doses |
|  | **origin** | Domestic | Domestic | **origin** | Imported | Domestic | **origin** | Imported | Imported |
|  | **Choice question:** |  |  | **Choice question:** |  |  | **Choice question:** |  |  |
| **Question 3** |  | **vaccine1** | **vaccine2** |  | **vaccine1** | **vaccine2** |  | **vaccine1** | **vaccine2** |
|  | **price** | 0 | 400 | **price** | 600 | 0 | **price** | 400 | 400 |
|  | **risk** | 0 | 1/100000 | **risk** | 0 | 1/100000 | **risk** | 1/100000 | 0 |
|  | **duration** | Life long | 6 months | **duration** | 12 months | 6 months | **duration** | 6 months | 12 months |
|  | **efficacy** | 90% | 70% | **efficacy** | 50% | 50% | **efficacy** | 70% | 90% |
|  | **admin** | Injection | Injection | **admin** | Injection | Oral | **admin** | Injection | Injection |
|  | **doses** | 1 dose | 2 doses | **doses** | 1 dose | 3 doses | **doses** | 2 doses | 3 doses |
|  | **origin** | Domestic | Imported | **origin** | Domestic | Imported | **origin** | Imported | Imported |
|  | **Choice question:** |  |  | **Choice question:** |  |  | **Choice question:** |  |  |
| **Question 4** |  | **vaccine1** | **vaccine2** |  | **vaccine1** | **vaccine2** |  | **vaccine1** | **vaccine2** |
|  | **price** | 0 | 200 | **price** | 200 | 600 | **price** | 200 | 200 |
|  | **risk** | 0 | 0 | **risk** | 1/1000000 | 0 | **risk** | 0 | 1/1000000 |
|  | **duration** | 6 months | life long | **duration** | 6 months | 6 months | **duration** | Life long | 6 months |
|  | **efficacy** | 50% | 50% | **efficacy** | 90% | 70% | **efficacy** | 50% | 70% |
|  | **admin** | Injection | Oral | **admin** | Oral | Oral | **admin** | Oral | Oral |
|  | **doses** | 3 doses | 3 doses | **doses** | 1 dose | 2 doses | **doses** | 3 doses | 1 dose |
|  | **origin** | Imported | Imported | **origin** | Domestic | Imported | **origin** | Imported | Imported |
|  | **Choice question:** |  |  | **Choice question:** |  |  | **Choice question:** |  |  |
| **Question 5** |  | **vaccine1** | **vaccine2** |  | **vaccine1** | **vaccine2** |  | **vaccine1** | **vaccine2** |
|  | **price** | 600 | 600 | **price** | 400 | 0 | **price** | 400 | 400 |
|  | **risk** | 1/1000000 | 1/100000 | **risk** | 1/1000000 | 1/1000000 | **risk** | 0 | 1/1000000 |
|  | **duration** | 12 months | 12 months | **duration** | Life long | 6 months | **duration** | 6 months | 12 months |
|  | **efficacy** | 90% | 50% | **efficacy** | 50% | 50% | **efficacy** | 90% | 50% |
|  | **admin** | Oral | Injection | **admin** | Oral | Injection | **admin** | Oral | Oral |
|  | **doses** | 3 doses | 1 dose | **doses** | 3 doses | 2 doses | **doses** | 1 dose | 2 doses |
|  | **origin** | Imported | Imported | **origin** | Domestic | Domestic | **origin** | Domestic | Domestic |
|  | **Choice question:** |  |  | **Choice question:** |  |  | **Choice question:** |  |  |
| **Question 6** |  | **vaccine1** | **vaccine2** |  | **vaccine1** | **vaccine2** |  | **vaccine1** | **vaccine2** |
|  | **price** | 0 | 600 | **price** | 600 | 400 | **price** | 0 | 200 |
|  | **risk** | 0 | 1/1000000 | **risk** | 1/1000000 | 1/1000000 | **risk** | 1/100000 | 1/100000 |
|  | **duration** | 12 months | 6 months | **duration** | Life long | life long | **duration** | 6 months | 12 months |
|  | **efficacy** | 70% | 90% | **efficacy** | 70% | 50% | **efficacy** | 50% | 90% |
|  | **admin** | Oral | Injection | **admin** | Injection | Oral | **admin** | Oral | Oral |
|  | **doses** | 1 dose | 3 doses | **doses** | 2 doses | 3 doses | **doses** | 3 doses | 2 doses |
|  | **origin** | Imported | Imported | **origin** | Domestic | Domestic | **origin** | Imported | Imported |
|  | **Choice question:** |  |  | **Choice question:** |  |  | **Choice question:** |  |  |
| **Question 7** |  | **vaccine1** | **vaccine2** |  | **vaccine1** | **vaccine2** |  | **vaccine1** | **vaccine2** |
|  | **price** | 0 | 400 | **price** | 200 | 0 | **price** | 200 | 600 |
|  | **risk** | 1/1000000 | 1/100000 | **risk** | 1/100000 | 1/1000000 | **risk** | 1/100000 | 0 |
|  | **duration** | 12 months | life long | **duration** | 12 months | 12 months | **duration** | Life long | life long |
|  | **efficacy** | 70% | 70% | **efficacy** | 50% | 70% | **efficacy** | 90% | 70% |
|  | **admin** | Injection | Oral | **admin** | Oral | Injection | **admin** | Injection | Injection |
|  | **doses** | 1 dose | 3 doses | **doses** | 2 doses | 1 dose | **doses** | 1 dose | 2 doses |
|  | **origin** | Imported | Domestic | **origin** | Domestic | Imported | **origin** | Domestic | Imported |
|  | **Choice question:** |  |  | **Choice question:** |  |  | **Choice question:** |  |  |
| **Question 8** |  | **vaccine1** | **vaccine2** |  | **vaccine1** | **vaccine2** |  | **vaccine1** | **vaccine2** |
|  | **price** | 200 | 0 | **price** | 400 | 600 | **price** | 600 | 600 |
|  | **risk** | 0 | 1/1000000 | **risk** | 1/1000000 | 1/100000 | **risk** | 0 | 1/1000000 |
|  | **duration** | 6 months | life long | **duration** | 12 months | life long | **duration** | Life long | life long |
|  | **efficacy** | 50% | 90% | **efficacy** | 50% | 50% | **efficacy** | 70% | 70% |
|  | **admin** | Injection | Oral | **admin** | Oral | Oral | **admin** | Injection | Injection |
|  | **doses** | 2 doses | 2 doses | **doses** | 2 doses | 1 dose | **doses** | 2 doses | 2 doses |
|  | **origin** | Domestic | Imported | **origin** | Domestic | Imported | **origin** | Imported | Domestic |
|  | **Choice question:** |  |  | **Choice question:** |  |  | **Choice question:** |  |  |
| **Question 9** |  | **vaccine1** | **vaccine2** |  | **vaccine1** | **vaccine2** |  | **vaccine1** | **vaccine2** |
|  | **price** | 600 | 0 | **price** | 0 | 0 | **price** | 400 | 400 |
|  | **risk** | 1/100000 | 1/100000 | **risk** | 1/1000000 | 1/100000 | **risk** | 0 | 1/1000000 |
|  | **duration** | Life long | 12 months | **duration** | 6 months | life long | **duration** | 12 months | life long |
|  | **efficacy** | 50% | 70% | **efficacy** | 50% | 90% | **efficacy** | 90% | 50% |
|  | **admin** | Oral | Injection | **admin** | Injection | Injection | **admin** | Injection | Injection |
|  | **doses** | 1 dose | 3 doses | **doses** | 2 doses | 2 doses | **doses** | 3 doses | 1 dose |
|  | **origin** | Imported | Domestic | **origin** | Domestic | Imported | **origin** | Imported | Imported |
|  | **Choice question:** |  |  | **Choice question:** |  |  | **Choice question:** |  |  |
| **Question 10** |  | **vaccine1** | **vaccine2** |  | **vaccine1** | **vaccine2** |  | **vaccine1** | **vaccine2** |
|  | **price** | 600 | 200 | **price** | 600 | 400 | **price** | 200 | 200 |
|  | **risk** | 1/100000 | 0 | **risk** | 1/1000000 | 0 | **risk** | 1/1000000 | 1/1000000 |
|  | **duration** | 6 months | 6 months | **duration** | 6 months | 12 months | **duration** | 6 months | 6 months |
|  | **efficacy** | 90% | 50% | **efficacy** | 90% | 90% | **efficacy** | 70% | 90% |
|  | **admin** | Injection | Injection | **admin** | Injection | Oral | **admin** | Oral | Oral |
|  | **doses** | 3 doses | 2 doses | **doses** | 3 doses | 2 doses | **doses** | 1 dose | 1 dose |
|  | **origin** | Domestic | Domestic | **origin** | Imported | Domestic | **origin** | Imported | Domestic |
|  | **Choice question:** |  |  | **Choice question:** |  |  | **Choice question:** |  |  |
| **Question 11** |  | **vaccine1** | **vaccine2** |  | **vaccine1** | **vaccine2** |  | **vaccine1** | **vaccine2** |
|  | **price** | 200 | 0 | **price** | 400 | 600 | **price** | 400 | 600 |
|  | **risk** | 1/100000 | 0 | **risk** | 1/1000000 | 1/1000000 | **risk** | 1/100000 | 1/100000 |
|  | **duration** | 12 months | 12 months | **duration** | Life long | 12 months | **duration** | 6 months | 6 months |
|  | **efficacy** | 90% | 70% | **efficacy** | 50% | 90% | **efficacy** | 70% | 90% |
|  | **admin** | Oral | Oral | **admin** | Injection | Oral | **admin** | Oral | Injection |
|  | **doses** | 2 doses | 1 dose | **doses** | 1 dose | 3 doses | **doses** | 1 dose | 3 doses |
|  | **origin** | Imported | Imported | **origin** | Imported | Imported | **origin** | Domestic | Domestic |
|  | **Choice question:** |  |  | **Choice question:** |  |  | **Choice question:** |  |  |
| **Question 12** |  | **vaccine1** | **vaccine2** |  | **vaccine1** | **vaccine2** |  | **vaccine1** | **vaccine2** |
|  | **price** | 400 | 600 | **price** | 400 | 200 | **price** | 200 | 400 |
|  | **risk** | 1/100000 | 0 | **risk** | 0 | 0 | **risk** | 0 | 1/100000 |
|  | **duration** | Life long | 12 months | **duration** | 12 months | life long | **duration** | Life long | 6 months |
|  | **efficacy** | 70% | 50% | **efficacy** | 90% | 70% | **efficacy** | 70% | 70% |
|  | **admin** | Oral | Injection | **admin** | Oral | Oral | **admin** | Oral | Oral |
|  | **doses** | 3 doses | 1 dose | **doses** | 2 doses | 3 doses | **doses** | 3 doses | 1 dose |
|  | **origin** | Domestic | Domestic | **origin** | Domestic | Domestic | **origin** | Domestic | Domestic |
|  | **Choice question:** |  |  | **Choice question:** |  |  | **Choice question:** |  |  |

**Part III: Basic personal information**

1. What is your age? (Unit: Years)

Please fill in numbers, decimals are allowed.

______________________________________

1. What is your weight? (Unit: kg)

Please fill in numbers, decimals are allowed.

______________________________________

1. What is your height? (Unit: cm)

Please fill in numbers, decimals are allowed.

______________________________________

1. What is your gender?

o Male (1)

o Female (2)

o Others (3)

1. Which province do you live in now?

Hebei Province, Shanxi Province, Liaoning Province, Jilin Province, Heilongjiang Province, Jiangsu Province, Zhejiang Province, Anhui Province, Fujian Province, Jiangxi Province, Shandong Province, Henan Province, Hubei Province, Hunan Province, Guangdong Province, Hainan Province, Sichuan Province , Guizhou Province, Yunnan Province, Shaanxi Province, Gansu Province, Qinghai Province, Inner Mongolia Autonomous Region, Guangxi Zhuang Autonomous Region, Tibet Autonomous Region, Ningxia Hui Autonomous Region, Xinjiang Uygur Autonomous Region, Beijing, Shanghai, Tianjin, Chongqing

1. What is your ethnicity?

□Han □Hui □Zang □Zhuang □Man □Others, please specify

1. Do you live in an urban area or a rural area?

□ Urban□Rural

1. What is your highest degree? (If you are still studying at school, please select the highest degree you have completed)

□Have never attended school □Elementary school □Junior high school □High school/technical secondary school □College/undergraduate □Graduate and above

1. Are you currently married?

□ Married□Unmarried (including divorced and widowed)

1. Which of the following work situations are you currently in?

□ Work in a private setting (including freelancers)

□ Work in a public setting

□ No job (including students, unemployment, retirement and other reasons)

1. Are you currently a medical worker in contact with patients?

□ Yes□ No

1. What is the total annual income (RMB) of your family?

□< 30000 □ 30000-60000 □60000-90000 □90000-120000

□120000- 150000 □ 150000-200000 □200000+

1. Do you have medical insurance?

□ Yes□ No

*Condition: Q13 Choice: Yes (1)
Display Q14*

1. What is your medical insurance type?

o Basic Medical Insurance for Urban Employees(1)

o Basic medical insurance for urban and rural residents (including basic medical insurance for urban residents and new rural cooperative medical insurance) (2)

**Part IV: Health status and vaccination behavior**

1. Do you have a habit of smoking?

o Yes   (1)

o No, but used to be   (2)

o No, never   (3)

*Condition: Q15 Choice: No, but used to be (2)
Display Q15.1*

15.1. How long have you quit smoking?

o Less than three months   (1)

o Three months – One year  (2)

o More than one year   (3)

1. Do you have a habit of drinking?

o Yes   (1)

o No, but used to be   (2)

o No, never   (3)

*Condition: Q16 Choice: No, but used to be (2)
Display Q16.1*

16.1. How long have you quit drinking?

o Less than three months   (1)

o Three months – One year  (2)

o More than one year   (3)

1. Have you ever been diagnosed as a chronic disease patient by a doctor or other medical staff?

o Yes   (1)

o No   (2)

1. What kind of chronic disease have you been diagnosed by a doctor or other medical staff?

o Hypertension (1)

o Diabetes   (2)

o Asthma (3)

o Chronic lung diseases other than asthma (4)

o Chronic Arterial Disease (5)

o Chronic Heart Disease (6)

o Chronic kidney disease (7)

o Cancer (8)

o Others, please specify (9)

1. In the past two weeks, how many times have you been troubled by any of the following problems?

| No. | Conditions | Never | Several days | More than half of the time | Almost everyday |
| --- | --- | --- | --- | --- | --- |
| 1 | Unmotivated or uninterested in doing things | 0 | 1 | 2 | 3 |
| 2 | Feeling down, depressed or hopeless | 0 | 1 | 2 | 3 |
| 3 | Having difficulty falling asleep; sleeping restless or oversleeping | 0 | 1 | 2 | 3 |
| 4 | Feeling tired or inactive | 0 | 1 | 2 | 3 |
| 5 | Loss of appetite or eating too much | 0 | 1 | 2 | 3 |
| 6 | Feeling bad or like a failure, or is disappointed with myself and my family | 0 | 1 | 2 | 3 |
| 7 | Having difficulty focusing on things like reading a newspaper or watching TV, etc. | 0 | 1 | 2 | 3 |
| 8 | Move or speak so slowly that other people have noticed it; or oppositely—become more irritable or figdet around | 0 | 1 | 2 | 3 |
| 9 | I ’d rather die or hurt myself in some way. | 0 | 1 | 2 | 3 |

*Conditions: Q4 Select: Female ( 2 )****&****Q1 Fill> 18 
Display Q20*

1. Have you been/now pregnant?

o Yes   (1)

o No   (2)

1. Have you ever been diagnosed with COVID-19?

o Yes (1)

o No (2)

1. Has anyone of your family members, neighbors, colleagues, friends or other people you know been diagnosed with COVID-19?

o Family members (1)

o Friends (2)

o Neighbors (3)

o Colleagues (4)

o Other people I know (5)

1. Have you been vaccinated against the SARS-CoV-2 virus?

o Yes (1)

o No (2)

*Condition: Q23 choose (1)
Display 23.1-23.4 .*

23.1. How long ago did you get the first dose of the COVID-19 vaccine?

o Within 1 month (1)

o 1 month-3 months ago   (2)

o 3 months- 6 months ago   (3)

23.2. How many shots of the COVID-19 vaccine have you received so far?

o One dose   (1)

o Two doses   (2)

o Three doses or more   (3)

23.3. Where did you get the COVID-19 vaccine?

o Mass (temporary) vaccination sites   (1)

o Secondary and tertiary hospital   (2)

o Primary hospitals/community health center/village clinics   (3)

23.4. Do you go for vaccination by yourself or participate in group vaccination?

o Self-vaccinations   (1)

o Group vaccination   (2)

1. In the past three years, have you received other non-mandatory general vaccines before? (Such as flu vaccine, bird flu vaccine, rabies vaccine, HPV vaccine, chickenpox vaccine, cholera vaccine, tetanus vaccine, etc.)

o Yes (1)

o No (2)

1. Please choose according to your degree of agreement with the following expressions, the number from 1 to 5 indicates that the degree of agreement increases

|  |  | strongly disagree | disagree | neutral | agree | strongly agree |
| --- | --- | --- | --- | --- | --- | --- |
| 1 | If I contact the new coronavirus, my health will be seriously damaged | 1 | 2 | 3 | 4 | 5 |
| 2 | I think the new coronavirus is more serious than the flu | 1 | 2 | 3 | 4 | 5 |
| 3 | Even if I have another disease, I will not go to the hospital because I am at risk of contracting the new coronavirus in the hospital | 1 | 2 | 3 | 4 | 5 |
| 4 | The new coronavirus will cause serious damage to my community | 1 | 2 | 3 | 4 | 5 |
| 5 | I am more susceptible to the new coronavirus than others | 1 | 2 | 3 | 4 | 5 |
| 6 | I believe I can resist the new coronavirus | 1 | 2 | 3 | 4 | 5 |
| 7 | I believe I can protect myself from the new coronavirus better than others | 1 | 2 | 3 | 4 | 5 |
| 8 | I am afraid of the new coronavirus | 1 | 2 | 3 | 4 | 5 |
| 9 | The new coronavirus has severely damaged the economy | 1 | 2 | 3 | 4 | 5 |

## Chinese version

新冠肺炎疫苗接种意愿调查问卷

**第一部分：简介**

当前，新冠病毒仍然在全球范围内肆虐。政府已经采取了针对新冠肺炎的疫苗接种项目。现在我们希望知道您认为我们应该接种疫苗应该有哪些属性以及您的偏好。这份问卷由五个部分组成：简介，选择实验，基本个人信息，健康状态与疫苗接种行为。

为了进一步研究需要，我们可能会在问卷中您的个人信息进行采集，但将对您的个人信息采取完全匿名处理，感谢您的支持与配合！

**第二部分：选择实验**

在下面的实验中，您将会看到12个不同的疫苗选择问题。请说明您在每个情形下可能做出的选择（疫苗一，疫苗二，或者不接种疫苗）。

请仔细阅读我们给出的信息，因为每个情形下疫苗信息都会发生改变。阅读信息，然后基于信息做出您的选择，选择无对错之分，我们关注的是您在每种情景下的想法和意见。

**属性说明**：

价格：接种的总费用;免费/200元/400元/600元

风险：接种后引发较严重副作用（如血栓，急性过敏反应）的概率；0 /百万分之一/十万分之一

保护期：疫苗接种后，疫苗将会保持预定效力的期限（超过保护期后效力可能会下降）；半年/一年/终身

保护效力：与未接种人群相比，接种后的易感人群受病毒感染概率下降的比例；50%，70%，90%

疫苗接种方式：注射/口服

接种频率：需要接种或口服的总次数；1次/2次/3次

疫苗产地：进口/国产

|  | **组1** |  |  | **组2** |  |  | **组3** |  |  |
| --- | --- | --- | --- | --- | --- | --- | --- | --- | --- |
| **问题1** |  | **疫苗1** | **疫苗2** |  | **疫苗1** | **疫苗2** |  | **疫苗1** | **疫苗2** |
|  | **价格** | 0 | 400 | **价格** | 600 | 200 | **价格** | 0 | 200 |
|  | **风险** | 1/1000000 | 0 | **风险** | 0 | 1/100000 | **风险** | 1/100000 | 1/1000000 |
|  | **保护期** | 终身 | 6个月 | **保护期** | 6个月 | 12个月 | **保护期** | 12个月 | 12个月 |
|  | **保护效力** | 90% | 90% | **保护效力** | 70% | 50% | **保护效力** | 70% | 70% |
|  | **疫苗接种方式** | 口服 | 口服 | **疫苗接种方式** | 口服 | 口服 | **疫苗接种方式** | 注射 | 注射 |
|  | **接种频率** | 2 次 | 1 次 | **接种频率** | 2 次 | 2 次 | **接种频率** | 3 次 | 3 次 |
|  | **疫苗产地** | 进口 | 国产 | **疫苗产地** | 进口 | 国产 | **疫苗产地** | 国产 | 国产 |
|  | **选择:** |  |  | **选择:** |  |  | **选择:** |  |  |
| **问题2** |  | **疫苗1** | **疫苗2** |  | **疫苗1** | **疫苗2** |  | **疫苗1** | **疫苗2** |
|  | **价格** | 200 | 200 | **价格** | 600 | 0 | **价格** | 0 | 0 |
|  | **风险** | 1/1000000 | 1/100000 | **风险** | 1/100000 | 0 | **风险** | 1/100000 | 0 |
|  | **保护期** | 12个月 | 终身 | **保护期** | 12个月 | 终身 | **保护期** | 终身 | 6个月 |
|  | **保护效力** | 70% | 90% | **保护效力** | 50% | 90% | **保护效力** | 90% | 50% |
|  | **疫苗接种方式** | 注射 | 注射 | **疫苗接种方式** | 注射 | 注射 | **疫苗接种方式** | 注射 | 注射 |
|  | **接种频率** | 3 次 | 1 次 | **接种频率** | 1 次 | 1 次 | **接种频率** | 2 次 | 3 次 |
|  | **疫苗产地** | 国产 | 国产 | **疫苗产地** | 进口 | 国产 | **疫苗产地** | 进口 | 进口 |
|  | **选择:** |  |  | **选择:** |  |  | **选择:** |  |  |
| **问题3** |  | **疫苗1** | **疫苗2** |  | **疫苗1** | **疫苗2** |  | **疫苗1** | **疫苗2** |
|  | **价格** | 0 | 400 | **价格** | 600 | 0 | **价格** | 400 | 400 |
|  | **风险** | 0 | 1/100000 | **风险** | 0 | 1/100000 | **风险** | 1/100000 | 0 |
|  | **保护期** | 终身 | 6个月 | **保护期** | 12个月 | 6个月 | **保护期** | 6个月 | 12个月 |
|  | **保护效力** | 90% | 70% | **保护效力** | 50% | 50% | **保护效力** | 70% | 90% |
|  | **疫苗接种方式** | 注射 | 注射 | **疫苗接种方式** | 注射 | 口服 | **疫苗接种方式** | 注射 | 注射 |
|  | **接种频率** | 1 次 | 2 次 | **接种频率** | 1 次 | 3 次 | **接种频率** | 2 次 | 3 次 |
|  | **疫苗产地** | 国产 | 进口 | **疫苗产地** | 国产 | 进口 | **疫苗产地** | 进口 | 进口 |
|  | **选择:** |  |  | **选择:** |  |  | **选择:** |  |  |
| **问题4** |  | **疫苗1** | **疫苗2** |  | **疫苗1** | **疫苗2** |  | **疫苗1** | **疫苗2** |
|  | **价格** | 0 | 200 | **价格** | 200 | 600 | **价格** | 200 | 200 |
|  | **风险** | 0 | 0 | **风险** | 1/1000000 | 0 | **风险** | 0 | 1/1000000 |
|  | **保护期** | 6个月 | 终身 | **保护期** | 6个月 | 6个月 | **保护期** | 终身 | 6个月 |
|  | **保护效力** | 50% | 50% | **保护效力** | 90% | 70% | **保护效力** | 50% | 70% |
|  | **疫苗接种方式** | 注射 | 口服 | **疫苗接种方式** | 口服 | 口服 | **疫苗接种方式** | 口服 | 口服 |
|  | **接种频率** | 3 次 | 3 次 | **接种频率** | 1 次 | 2 次 | **接种频率** | 3 次 | 1 次 |
|  | **疫苗产地** | 进口 | 进口 | **疫苗产地** | 国产 | 进口 | **疫苗产地** | 进口 | 进口 |
|  | **选择:** |  |  | **选择:** |  |  | **选择:** |  |  |
| **问题5** |  | **疫苗1** | **疫苗2** |  | **疫苗1** | **疫苗2** |  | **疫苗1** | **疫苗2** |
|  | **价格** | 600 | 600 | **价格** | 400 | 0 | **价格** | 400 | 400 |
|  | **风险** | 1/1000000 | 1/100000 | **风险** | 1/1000000 | 1/1000000 | **风险** | 0 | 1/1000000 |
|  | **保护期** | 12个月 | 12个月 | **保护期** | 终身 | 6个月 | **保护期** | 6个月 | 12个月 |
|  | **保护效力** | 90% | 50% | **保护效力** | 50% | 50% | **保护效力** | 90% | 50% |
|  | **疫苗接种方式** | 口服 | 注射 | **疫苗接种方式** | 口服 | 注射 | **疫苗接种方式** | 口服 | 口服 |
|  | **接种频率** | 3 次 | 1 次 | **接种频率** | 3 次 | 2 次 | **接种频率** | 1 次 | 2 次 |
|  | **疫苗产地** | 进口 | 进口 | **疫苗产地** | 国产 | 国产 | **疫苗产地** | 国产 | 国产 |
|  | **选择:** |  |  | **选择:** |  |  | **选择:** |  |  |
| **问题6** |  | **疫苗1** | **疫苗2** |  | **疫苗1** | **疫苗2** |  | **疫苗1** | **疫苗2** |
|  | **价格** | 0 | 600 | **价格** | 600 | 400 | **价格** | 0 | 200 |
|  | **风险** | 0 | 1/1000000 | **风险** | 1/1000000 | 1/1000000 | **风险** | 1/100000 | 1/100000 |
|  | **保护期** | 12个月 | 6个月 | **保护期** | 终身 | 终身 | **保护期** | 6个月 | 12个月 |
|  | **保护效力** | 70% | 90% | **保护效力** | 70% | 50% | **保护效力** | 50% | 90% |
|  | **疫苗接种方式** | 口服 | 注射 | **疫苗接种方式** | 注射 | 口服 | **疫苗接种方式** | 口服 | 口服 |
|  | **接种频率** | 1 次 | 3 次 | **接种频率** | 2 次 | 3 次 | **接种频率** | 3 次 | 2 次 |
|  | **疫苗产地** | 进口 | 进口 | **疫苗产地** | 国产 | 国产 | **疫苗产地** | 进口 | 进口 |
|  | **选择:** |  |  | **选择:** |  |  | **选择:** |  |  |
| **问题7** |  | **疫苗1** | **疫苗2** |  | **疫苗1** | **疫苗2** |  | **疫苗1** | **疫苗2** |
|  | **价格** | 0 | 400 | **价格** | 200 | 0 | **价格** | 200 | 600 |
|  | **风险** | 1/1000000 | 1/100000 | **风险** | 1/100000 | 1/1000000 | **风险** | 1/100000 | 0 |
|  | **保护期** | 12个月 | 终身 | **保护期** | 12个月 | 12个月 | **保护期** | 终身 | 终身 |
|  | **保护效力** | 70% | 70% | **保护效力** | 50% | 70% | **保护效力** | 90% | 70% |
|  | **疫苗接种方式** | 注射 | 口服 | **疫苗接种方式** | 口服 | 注射 | **疫苗接种方式** | 注射 | 注射 |
|  | **接种频率** | 1 次 | 3 次 | **接种频率** | 2 次 | 1 次 | **接种频率** | 1 次 | 2 次 |
|  | **疫苗产地** | 进口 | 国产 | **疫苗产地** | 国产 | 进口 | **疫苗产地** | 国产 | 进口 |
|  | **选择:** |  |  | **选择:** |  |  | **选择:** |  |  |
| **问题8** |  | **疫苗1** | **疫苗2** |  | **疫苗1** | **疫苗2** |  | **疫苗1** | **疫苗2** |
|  | **价格** | 200 | 0 | **价格** | 400 | 600 | **价格** | 600 | 600 |
|  | **风险** | 0 | 1/1000000 | **风险** | 1/1000000 | 1/100000 | **风险** | 0 | 1/1000000 |
|  | **保护期** | 6个月 | 终身 | **保护期** | 12个月 | 终身 | **保护期** | 终身 | 终身 |
|  | **保护效力** | 50% | 90% | **保护效力** | 50% | 50% | **保护效力** | 70% | 70% |
|  | **疫苗接种方式** | 注射 | 口服 | **疫苗接种方式** | 口服 | 口服 | **疫苗接种方式** | 注射 | 注射 |
|  | **接种频率** | 2 次 | 2 次 | **接种频率** | 2 次 | 1 次 | **接种频率** | 2 次 | 2 次 |
|  | **疫苗产地** | 国产 | 进口 | **疫苗产地** | 国产 | 进口 | **疫苗产地** | 进口 | 国产 |
|  | **选择:** |  |  | **选择:** |  |  | **选择:** |  |  |
| **问题9** |  | **疫苗1** | **疫苗2** |  | **疫苗1** | **疫苗2** |  | **疫苗1** | **疫苗2** |
|  | **价格** | 600 | 0 | **价格** | 0 | 0 | **价格** | 400 | 400 |
|  | **风险** | 1/100000 | 1/100000 | **风险** | 1/1000000 | 1/100000 | **风险** | 0 | 1/1000000 |
|  | **保护期** | 终身 | 12个月 | **保护期** | 6个月 | 终身 | **保护期** | 12个月 | 终身 |
|  | **保护效力** | 50% | 70% | **保护效力** | 50% | 90% | **保护效力** | 90% | 50% |
|  | **疫苗接种方式** | 口服 | 注射 | **疫苗接种方式** | 注射 | 注射 | **疫苗接种方式** | 注射 | 注射 |
|  | **接种频率** | 1 次 | 3 次 | **接种频率** | 2 次 | 2 次 | **接种频率** | 3 次 | 1 次 |
|  | **疫苗产地** | 进口 | 国产 | **疫苗产地** | 国产 | 进口 | **疫苗产地** | 进口 | 进口 |
|  | **选择:** |  |  | **选择:** |  |  | **选择:** |  |  |
| **问题10** |  | **疫苗1** | **疫苗2** |  | **疫苗1** | **疫苗2** |  | **疫苗1** | **疫苗2** |
|  | **价格** | 600 | 200 | **价格** | 600 | 400 | **价格** | 200 | 200 |
|  | **风险** | 1/100000 | 0 | **风险** | 1/1000000 | 0 | **风险** | 1/1000000 | 1/1000000 |
|  | **保护期** | 6个月 | 6个月 | **保护期** | 6个月 | 12个月 | **保护期** | 6个月 | 6个月 |
|  | **保护效力** | 90% | 50% | **保护效力** | 90% | 90% | **保护效力** | 70% | 90% |
|  | **疫苗接种方式** | 注射 | 注射 | **疫苗接种方式** | 注射 | 口服 | **疫苗接种方式** | 口服 | 口服 |
|  | **接种频率** | 3 次 | 2 次 | **接种频率** | 3 次 | 2 次 | **接种频率** | 1 次 | 1 次 |
|  | **疫苗产地** | 国产 | 国产 | **疫苗产地** | 进口 | 国产 | **疫苗产地** | 进口 | 国产 |
|  | **选择:** |  |  | **选择:** |  |  | **选择:** |  |  |
| **问题11** |  | **疫苗1** | **疫苗2** |  | **疫苗1** | **疫苗2** |  | **疫苗1** | **疫苗2** |
|  | **价格** | 200 | 0 | **价格** | 400 | 600 | **价格** | 400 | 600 |
|  | **风险** | 1/100000 | 0 | **风险** | 1/1000000 | 1/1000000 | **风险** | 1/100000 | 1/100000 |
|  | **保护期** | 12个月 | 12个月 | **保护期** | 终身 | 12个月 | **保护期** | 6个月 | 6个月 |
|  | **保护效力** | 90% | 70% | **保护效力** | 50% | 90% | **保护效力** | 70% | 90% |
|  | **疫苗接种方式** | 口服 | 口服 | **疫苗接种方式** | 注射 | 口服 | **疫苗接种方式** | 口服 | 注射 |
|  | **接种频率** | 2 次 | 1 次 | **接种频率** | 1 次 | 3 次 | **接种频率** | 1 次 | 3 次 |
|  | **疫苗产地** | 进口 | 进口 | **疫苗产地** | 进口 | 进口 | **疫苗产地** | 国产 | 国产 |
|  | **选择:** |  |  | **选择:** |  |  | **选择:** |  |  |
| **问题12** |  | **疫苗1** | **疫苗2** |  | **疫苗1** | **疫苗2** |  | **疫苗1** | **疫苗2** |
|  | **价格** | 400 | 600 | **价格** | 400 | 200 | **价格** | 200 | 400 |
|  | **风险** | 1/100000 | 0 | **风险** | 0 | 0 | **风险** | 0 | 1/100000 |
|  | **保护期** | 终身 | 12个月 | **保护期** | 12个月 | 终身 | **保护期** | 终身 | 6个月 |
|  | **保护效力** | 70% | 50% | **保护效力** | 90% | 70% | **保护效力** | 70% | 70% |
|  | **疫苗接种方式** | 口服 | 注射 | **疫苗接种方式** | 口服 | 口服 | **疫苗接种方式** | 口服 | 口服 |
|  | **接种频率** | 3 次 | 1 次 | **接种频率** | 2 次 | 3 次 | **接种频率** | 3 次 | 1 次 |
|  | **疫苗产地** | 国产 | 国产 | **疫苗产地** | 国产 | 国产 | **疫苗产地** | 国产 | 国产 |
|  | **选择:** |  |  | **选择:** |  |  | **选择:** |  |  |

**第三部分：个人基本信息**

1. 您的年龄是？（单位：岁）

请填写数字，允许填写小数。

1. ______________________________________
2. 您的体重为？（单位：公斤）
3. 请填写数字，允许填写小数。
4. ______________________________________
5. 您的身高为？（单位：厘米）
6. 请填写数字，允许填写小数。
7. ______________________________________
8. 您的性别是？

o 男（1）

o 女（2）

o 其他（3）

1. 您现在居住在哪个省份？

河北省、山西省、辽宁省、吉林省、黑龙江省、江苏省、浙江省、安徽省、福建省、江西省、山东省、河南省、湖北省、湖南省、广东省、海南省、四川省、贵州省、云南省、陕西省、甘肃省、青海省、内蒙古自治区、广西壮族自治区、西藏自治区、宁夏回族自治区、新疆维吾尔自治区、北京市、上海市、天津市、重庆市

1. 你的民族是？

□汉族 □回族 □藏族□壮族□满族 □其他，请说明

1. 请问您居住的是城镇还是农村？

□城镇 □农村

1. 您的最高学历是？（如果现在依然在校读书，请选择您已念完的最高学历）

□没上过学 □小学 □初中 □高中/中专 □大专/本科 □研究生及以上

1. 您目前是否已婚？

□已婚 □未婚(包含离异和丧偶)

1. 您目前属于以下哪种工作情形？

□在私人场所工作（含自由职业者）

□在公众场所工作

□没有工作（包括学生，失业，退休其他原因）

1. 您目前是与病人接触的医务工作者吗？

□是 □否

1. 您家庭总年收入（人民币）大约是多少？

□<30000 □30000-60000 □60000-90000 □90000-120000

□120000-150000 □150000-200000 □200000+

1. 您是否有医疗保险？

□是 □否

*条件：Q13 选择：是（1）*

*出现Q14*

1. 您的医疗保险类型是？

o城镇职工基本医疗保险

o城乡居民基本医疗保险（含城镇居民基本医疗保险、新型农村合作医疗保险）

**第四部分：健康状态及疫苗接种行为**

1. 您是否有吸烟的习惯？
2. o是 (1)
3. o否，但曾经是 (2)
4. o否，从来都不是 (3)

*条件: Q15 选择: 否, 从来都不是 (2)
出现 Q15.1*

15.1. 您是从多久之前开始戒烟的?

o 少于三个月   (1)

o 三个月至一年  (2)

o 超过一年   (3)

1. 您是否有喝酒的习惯？
2. o是 (1)
3. o否，但曾经是 (2)
4. o否，从来都不是 (3)

*条件: Q16 选择: 否, 从来都不是 (2)
出现 Q16.1*

16.1. 您是从多久之前开始戒酒的?

o 少于三个月   (1)

o 三个月至一年  (2)

o 超过一年   (3)

1. 您是否曾经被医生或其他医护人员诊断为慢性疾病患者？
2. o是 (1)
3. o否 (2)
4. 您曾经被医生或其他医护人员诊断为哪种慢性疾病患者？
5. O高血压 (1)
6. O糖尿病 (2)
7. O哮喘 (3)
8. O 哮喘以外的慢性肺部疾病 (4)
9. O 慢性动脉疾病 (5)
10. O 慢性心脏病 (6)
11. O 慢性肾脏疾病 (7)
12. O 癌症 (8)
13. 在过去两个星期，有多少时候您受到以下任何问题所困扰？

|  |  | 完全没有 | 几天 | 一半以上的天数 | 几乎每天 |
| --- | --- | --- | --- | --- | --- |
| 1 | 做事时提不起劲或没有兴趣 | 0 | 1 | 2 | 3 |
| 2 | 感到心情低落, 沮丧或绝望 | 0 | 1 | 2 | 3 |
| 3 | 入睡困难、睡不安或睡得过多 | 0 | 1 | 2 | 3 |
| 4 | 感觉疲倦或没有活力 | 0 | 1 | 2 | 3 |
| 5 | 食欲不振或吃太多 | 0 | 1 | 2 | 3 |
| 6 | 觉得自己很糟或觉得自己很失败，或让自己、家人失望 | 0 | 1 | 2 | 3 |
| 7 | 对事物专注有困难，例如看报纸或看电视时 | 0 | 1 | 2 | 3 |
| 8 | 行动或说话速度缓慢到别人已经察觉；或刚好相反——变得比平日更烦躁或坐立不安，动来动去 | 0 | 1 | 2 | 3 |
| 9 | 有不如死掉或用某种方式伤害自己的念头 | 0 | 1 | 2 | 3 |

*条件：Q4 选择：女（2）****并且****Q1填写：>18*

*出现Q20*

1. 您是否曾经/现在怀孕？
2. o是 (1)
3. o否 (2)
4. 您是否曾被诊断出患有新冠肺炎？

o是 (1)

o否 (2)

1. 您的家庭成员、邻居、同事、朋友或您认识的其他人中是否有人被诊断出患有新冠肺炎？

o家庭成员（1）

o朋友（2）

o邻居（3）

o同事（4）

o我认识的其他人（5）

1. 您是否已经接种过新冠肺炎疫苗？

o是 (1)

o否 (2)

*条件：Q23 选择：是（1），出现23.1-23.4.*

- 1. 您在多久之前接种过第一针新冠疫苗？

o 1个月之内 (1)

o 1个月-3个月前 (2)

o 3个月-6个月前 (3)

- 1. 您目前已经接种了几针新冠疫苗？

o 一针 (1)

o 两针 (2)

o 三针或以上 (3)

- 1. 您在哪里接种的新冠疫苗？

o 大型（临时）疫苗接种点 (1)

o 二级或三级医院 (2)

o 一级医院/社区医院/乡村诊所/卫生服务站 (3)

- 1. 您是自行前往接种还是参与集体接种？

o 自行接种 (1)

o 集体接种 (2)

1. 在过去三年内，您之前是否接种过其他非强制性普通疫苗？（如流感疫苗，禽流感疫苗，狂犬病疫苗，HPV疫苗，水痘疫苗，霍乱疫苗，破伤风疫苗等）

o是 (1)

o否 (2)

1. 请根据您对以下表述的赞同程度进行选择，数字从1到5表示赞同程度递增

|  |  | 非常不同意 | 不同意 | 中立 | 同意 | 非常同意 |
| --- | --- | --- | --- | --- | --- | --- |
| 1 | 如果我感染了新型冠状病毒，我的健康将受到严重损害 | 1 | 2 | 3 | 4 | 5 |
| 2 | 我认为新型冠状病毒比流感更严重 | 1 | 2 | 3 | 4 | 5 |
| 3 | 即使我患上另一种疾病，我也不会去医院，因为我有在医院感染新型冠状病毒的风险 | 1 | 2 | 3 | 4 | 5 |
| 4 | 新型冠状病毒将对我的社区造成严重损害 | 1 | 2 | 3 | 4 | 5 |
| 5 | 我比其他人更容易感染新型冠状病毒 | 1 | 2 | 3 | 4 | 5 |
| 6 | 我相信我能抵御新型冠状病毒 | 1 | 2 | 3 | 4 | 5 |
| 7 | 我相信我能比其他人更好地保护自己免受新型冠状病毒的侵害 | 1 | 2 | 3 | 4 | 5 |
| 8 | 我害怕新型冠状病毒 | 1 | 2 | 3 | 4 | 5 |
| 9 | 新型冠状病毒严重损害了经济 | 1 | 2 | 3 | 4 | 5 |
