# Supplementary material for: Preferences regarding COVID-19 vaccination among 12,000 adults in China: A cross-sectional discrete choice experiment
Source: PLOS Glob Public Health. 2024 Jul 11;4(7):e0003387. doi: 10.1371/journal.pgph.0003387 (PMC11239003; doi:10.1371/journal.pgph.0003387)
Supplement: S2 Text — (DOCX) [file pgph.0003387.s003.docx]

## S2 Text: Survey experiment development

### Selection of attributes

The analysis of COVID-19 vaccine attributes and corresponding levels drew on a sequential mixed-methods approach. In the first step, we conducted a comprehensive literature review of articles that employed a variety of research methods to investigate the preferences for different vaccine features and programs.^1-4^ From this review, we formed a candidate set of attributes, such as price, efficacy, duration of protection, delivery, etc., as well as levels for these attributes (e.g., 50% efficacy vs. 70% efficacy vs. 90% efficacy).

In the second step, we conducted in-depth qualitative online interviews with participants to identify key factors that affected their willingness to take up vaccines. These interviews allowed us to explore essential vaccine attributes in more detail and enabled a more robust analysis of our findings. Participants were randomly selected based on three demographic characteristics: age: old (≥ 50 years old) or young (> 18 years old and ＜ 50 years old), gender: male or female, and place of residence: rural or urban. We recruited five participants for each of the eight possible combinations of characteristics, resulting in 40 total participants. This recruitment strategy struck a balance between ensuring a broad spectrum of perspectives and limiting the total interviews to be conducted to a reasonable number. While five interviews for each combination might not have been enough to reach saturation, it was considered sufficient to identify recurrent themes based on our previous experience and guidance from the literature. Additionally, for each characteristic-specific respondent group (e.g., the 20 younger people vs. the 20 older people), the sample size was potentially sufficient to reach saturation. Having five participants per each of the eight possible combinations of characteristics (and 20 participants per each characteristic across combinations) was therefore deemed sufficient for achieving in-depth qualitative exploration to meaningfully inform further study phases while limiting the burden of extended interviews for both participants and the research team.

In the final step, seven attributes related to the COVID-19 vaccine were included within the discrete choice experiment (DCE) design based on the previous studies^5^ and semi-structured qualitative interviews: total price, risk of rare but serious side-effects from the vaccine, duration of protection, degree of efficacy, vaccine administration, frequency of vaccination and vaccination origin. Levels for all attributes are also derived from the qualitative results. Despite the vaccines being distributed free of charge during the survey, we included price as an attribute. The main reason is due to its significance in gauging respondents' willingness to pay for the vaccine. Price remains a critical factor influencing vaccine acceptance, even when it’s not directly borne by individuals. In our preliminary literature review and qualitative online interviews, price is always a crucial factor that influences respondents’ choices. Therefore, including price as an attribute provides valuable insights for policymakers to optimize future vaccination policies.

### DCE design

After specifying all the attributes and levels, we generated the hypothetical alternatives and combined them to create choice sets. Prior to the final DCE design, a pilot study recruited 45 adults with similar characteristics to the target population was conducted in June 2021, aimed to check the clarity of the vaccine attribute definitions, confirm the appropriateness of size of the choice sets, and generate prior parameters for the final design ^2 6^. The initial pilot design assumed all zero prior parameters. To test the internal consistency, we provided each participant with 12 choice sets, including a dominant scenario (Question 4) in which the dominant alternative was obviously preferred with respect to all attribute levels than the other. The result of the pilot suggested no cognitive difficulties in understanding the choice sets, and nearly all the participants passed the internal consistency test except for two of 45 participants who chose an opt-out option.

Using the parameter estimates derived from the pilot study as the prior, we continued establishing an experiment design to improve the estimation efficiency of our design. These seven attributes resulted in 1,264 hypothetical vaccine profiles (one attribute at four levels, four attributes at three levels, and two attributes at two levels=4*3^4^*2^2^), and a total of 839,160 possible pairwise choices ((1,296*1,295/2)). Considering that full factorial design was too tedious and costly in this case, the sequential orthogonal designs would typically lead to smaller designs in terms of the number of choice situations of the design^7^ and orthogonality guarantees that the effect of one factor or interaction can be estimated separately from other factors and interaction in the model^8^.

In the final design, we used Ngene version 1.2.1 DCE design software package to generate 36 choice sets based on the results from the pilot, among which no alternative dominated the other in each choice set. To relieve the cognitive burden of the participants, we grouped 36 choice sets into three blocks, and each contained 12 choice sets. **S1 Table** presents an example of choice tasks provided to the participants.

### Quality control

As a quality control measure, the participants must finish this survey between the time period of 360s and 1000s and verify understanding of the details before signing. In addition, we also checked the consistency of the survey data in age, marital status, and education status to exclude the participants with inconsistent responses.

## References

1. Poulos C. A Review of Discrete Choice Experiment Studies of Preferences for Vaccine Features. *Value in Health* 2016;19:A220. doi: 10.1016/j.jval.2016.03.1185

2. Abiiro GA, Torbica A, Kwalamasa K, et al. Eliciting community preferences for complementary micro health insurance: A discrete choice experiment in rural Malawi. *Social Science & Medicine* 2014;120:160-68. doi: <https://doi.org/10.1016/j.socscimed.2014.09.021>

3. Poulos C, Yang J-C, Levin C, et al. Mothers’ preferences and willingness to pay for HPV vaccines in Vinh Long Province, Vietnam. *Social Science & Medicine* 2011;73(2):226-34. doi: 10.1016/j.socscimed.2011.05.029

4. Harapan H, Wagner AL, Yufika A, et al. Willingness-to-pay for a COVID-19 vaccine and its associated determinants in Indonesia. *Human Vaccines & Immunotherapeutics* 2020;16(12):3074-80. doi: 10.1080/21645515.2020.1819741

5. Schwarzinger M, Watson V, Arwidson P, et al. COVID-19 vaccine hesitancy in a representative working-age population in France: a survey experiment based on vaccine characteristics. *The Lancet Public Health* 2021;6(4):e210-e21. doi: 10.1016/s2468-2667(21)00012-8

6. Wang Q, Abiiro GA, Yang J, et al. Preferences for long-term care insurance in China: Results from a discrete choice experiment. *Social Science & Medicine* 2021;281:114104. doi: <https://doi.org/10.1016/j.socscimed.2021.114104>

7. ChoiceMetrics. Ngene 1.1.1 User Manual & Reference Guide2012.

8. Marshall HS, Chen G, Clarke M, et al. Adolescent, parent and societal preferences and willingness to pay for meningococcal B vaccine: A Discrete Choice Experiment. *Vaccine* 2016;34(5):671-77. doi: 10.1016/j.vaccine.2015.11.075
