# Supplementary figures and images for: Preferences regarding COVID-19 vaccination among 12,000 adults in China: A cross-sectional discrete choice experiment
Source: PLOS Glob Public Health. 2024 Jul 11;4(7):e0003387. doi: 10.1371/journal.pgph.0003387 (PMC11239003; doi:10.1371/journal.pgph.0003387)

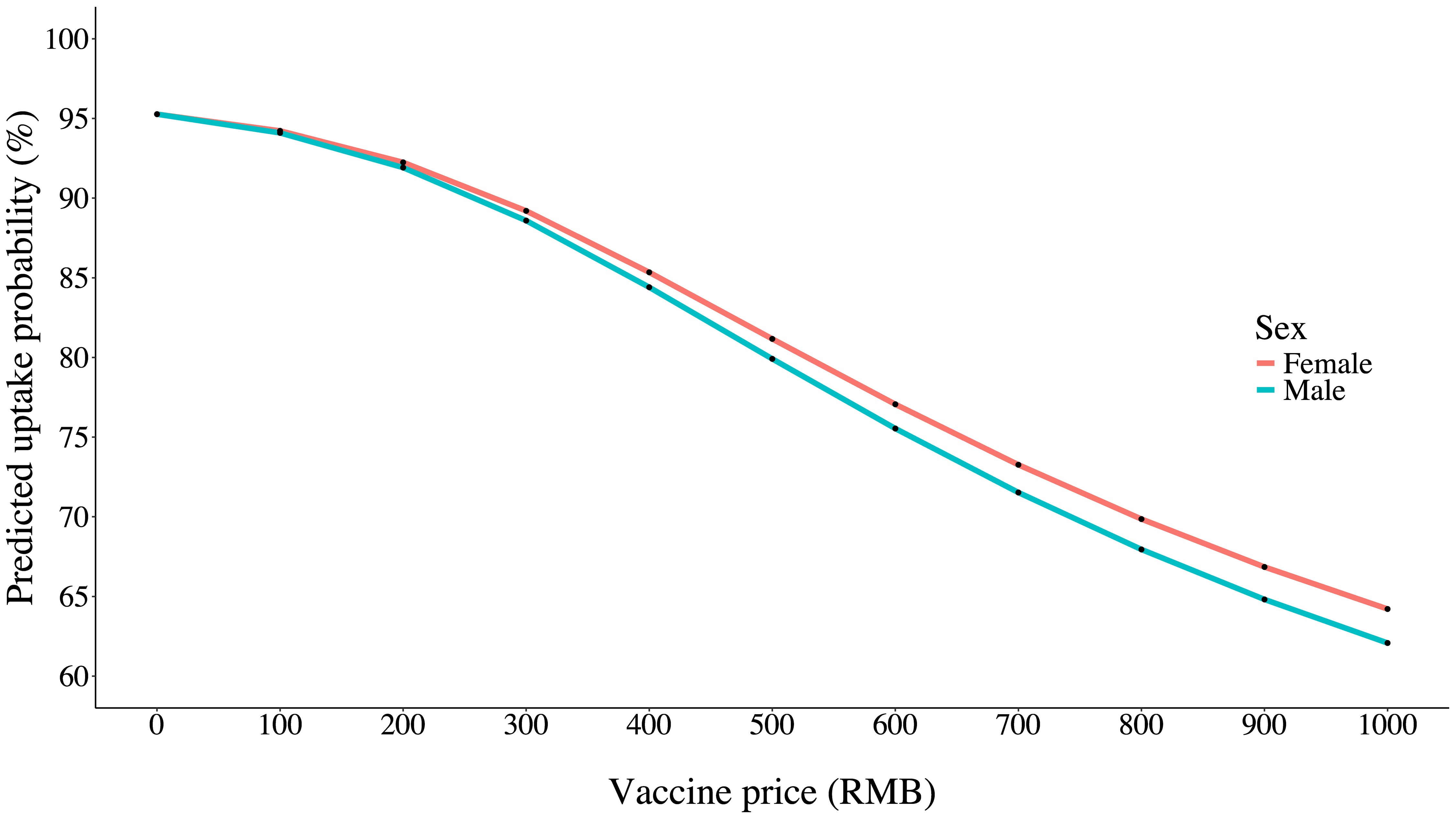

Supplement: S1 Fig — (TIFF) [file pgph.0003387.s004.tiff]

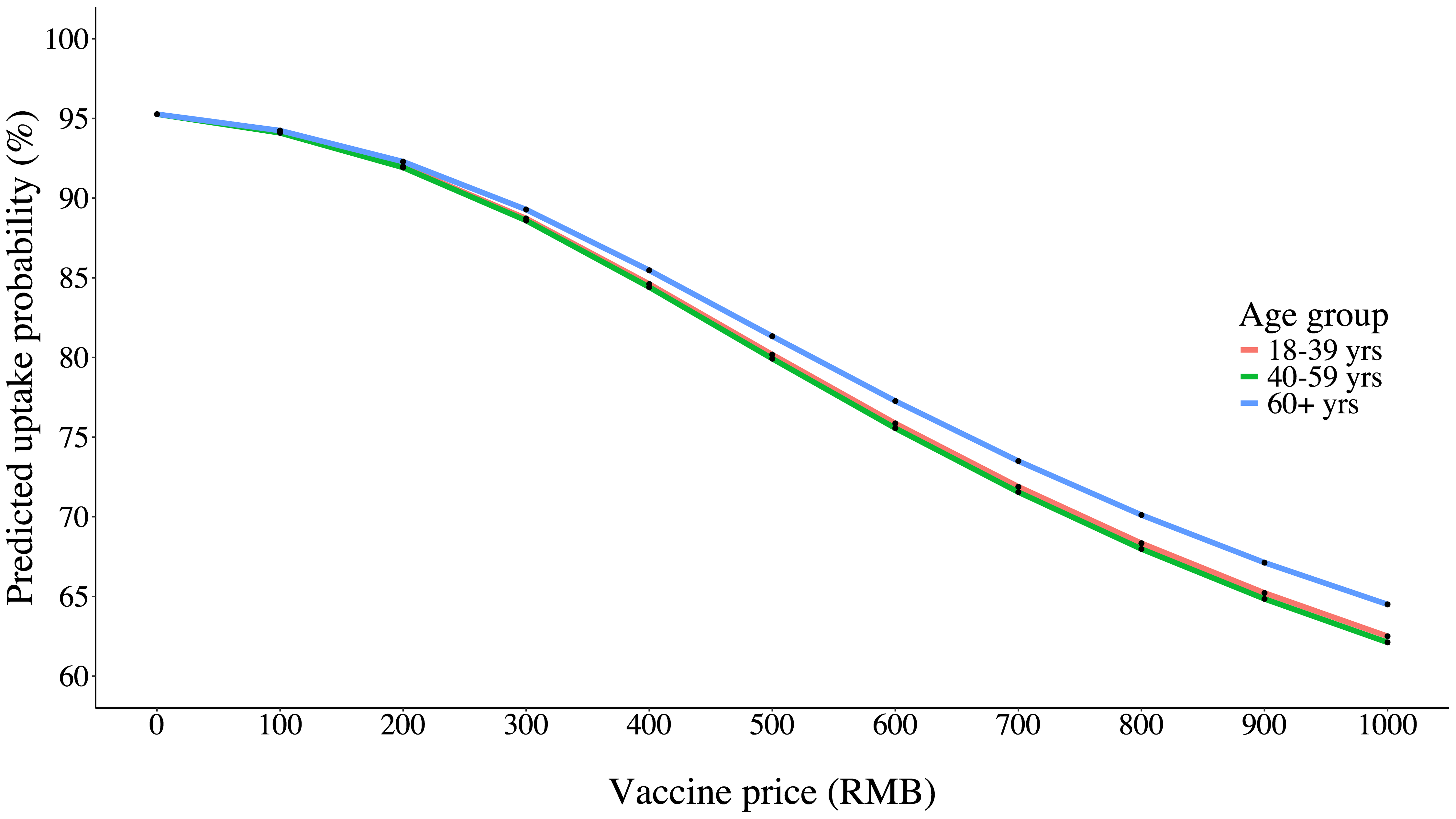

Supplement: S2 Fig — (TIFF) [file pgph.0003387.s005.tiff]

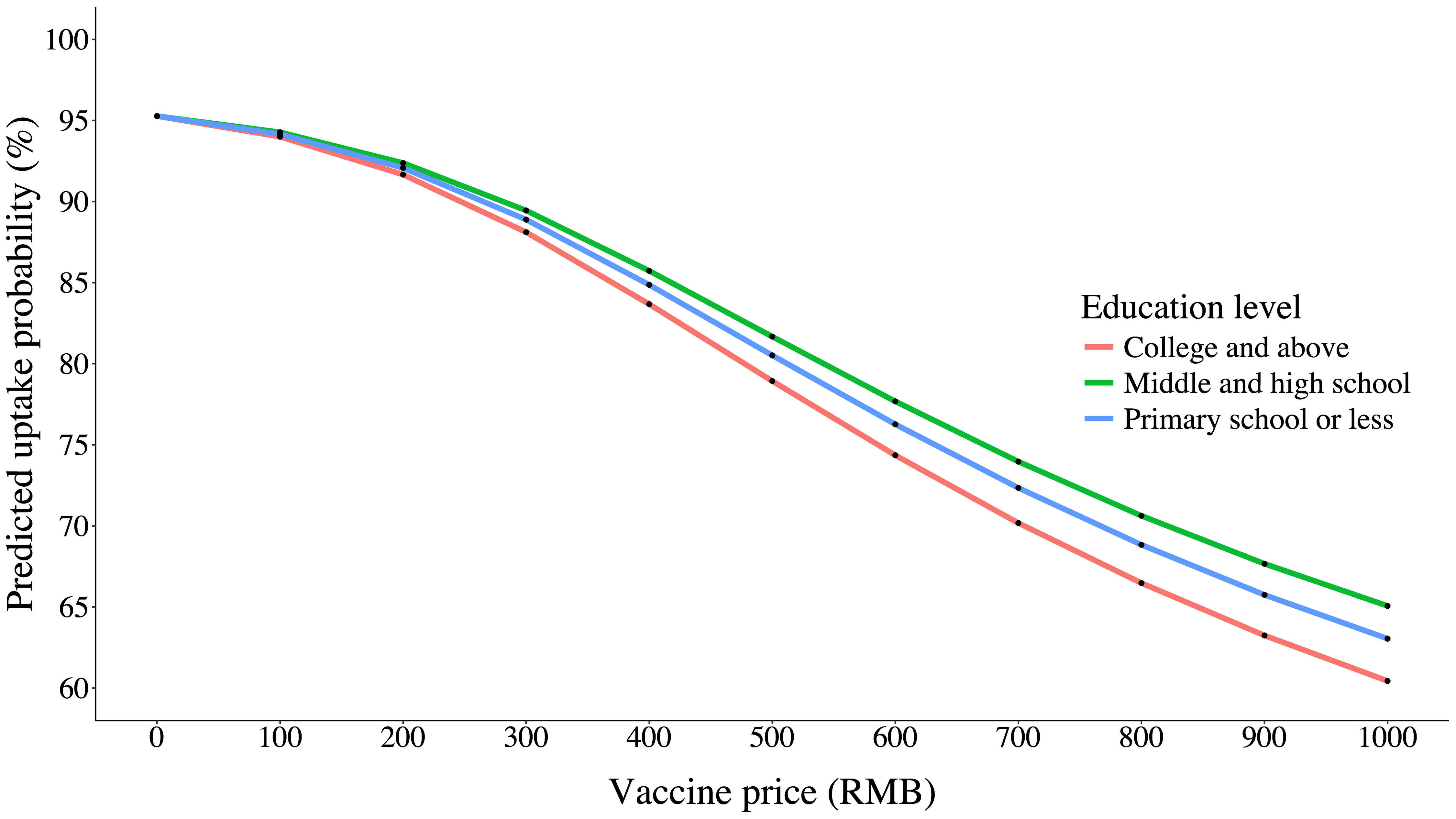

Supplement: S3 Fig — (TIFF) [file pgph.0003387.s006.tiff]
