# Supplementary material for: Preferences regarding COVID-19 vaccination among 12,000 adults in China: A cross-sectional discrete choice experiment
Source: PLOS Glob Public Health. 2024 Jul 11;4(7):e0003387. doi: 10.1371/journal.pgph.0003387 (PMC11239003; doi:10.1371/journal.pgph.0003387)
Supplement: S1 Table — (DOCX) [file pgph.0003387.s007.docx]

## S1 Table: Example of choice task

|  | **Vaccine 1** | **Vaccine 2** | **Neither vaccine** |
| --- | --- | --- | --- |
| **Total price** | Free | 200 |  |
| **Risk of rare but serious side-effects from the vaccine** | 1/100000 | 1/1000000 |  |
| **Duration of protection** | 12 months | life long |  |
| **Degree of efficacy** | 70% | 90% |  |
| **Vaccine administration** | Oral | Injection |  |
| **Frequency of vaccination** | 3 doses | 1 dose |  |
| **Vaccination origin** | Imported | Domestic |  |
| **Choice:** |  |  |  |
