# Supplementary material for: Preferences regarding COVID-19 vaccination among 12,000 adults in China: A cross-sectional discrete choice experiment
Source: PLOS Glob Public Health. 2024 Jul 11;4(7):e0003387. doi: 10.1371/journal.pgph.0003387 (PMC11239003; doi:10.1371/journal.pgph.0003387)
Supplement: S2 Table — (DOCX) [file pgph.0003387.s008.docx]

## S2 Table: Results of the basic mixed logit model

**Table S2** reports the main effects from the basic mixed logit model. The standard deviation of the vaccine attribute level “2 doses” was not significantly different from 0 (coefficient 0.010, [-0.136, 0.155]), which indicated that we do not need to include this attribute when adding the interaction in the extended mixed logit model.

**Table S2. Main effects from the basic mixed logit model**

| **Attribute and attribute levels** | | **Coefficient (**$\boldsymbol{\mu}_{\boldsymbol{m}}$**)^a^** | **95% CI of** $\boldsymbol{\mu}_{\boldsymbol{m}}$ | **SD (**$\boldsymbol{\delta}_{\boldsymbol{m}}$**)^b^** | **95% CI of** $\boldsymbol{\delta}_{\boldsymbol{m}}$ |
| --- | --- | --- | --- | --- | --- |
| ASC |  | 2.842 | [2.769, 2.914] | - | - |
| ASC (1st) |  | 0.123 | [0.099, 0.147] | - | - |
| Price (per 100 RMB) | | -0.166 | [-0.176, -0.166] | -0.379 | [-0.392, -0.366] |
| Risk |  |  |  |  |  |
|  | No risk | Ref |  |  |  |
|  | Moderate risk | -0.740 | [-0.780, -0.704] | 0.777 | [0.723, 0.831] |
|  | High risk | -0.769 | [-0.806, -0.730] | 0.718 | [0.662, 0.774] |
| Duration of protection | |  |  |  |  |
|  | 6 months | Ref |  | - | - |
|  | 12 months | 0.483 | [0.451, 0.515] | -0.331 | [-0.500, -0.162] |
|  | Life long | 1.638 | [1.590, 1.686] | 1.435 | [1.382, 1.488] |
| Degree of efficacy |  |  |  |  |  |
|  | 50% | Ref |  | - | - |
|  | 70% | 0.660 | [0.624, 0.697] | -0.434 | [-0.600, -0.269] |
|  | 90% | 1.452 | [1.409, 1.495] | -1.185 | [-1.231, -1.138] |
| Oral vaccine |  | -0.124 | [-0.154, -0.094] | -0.717 | [-0.780, -0.655] |
| Frequency of vaccination | |  |  |  |  |
|  | One dose | Ref |  | - | - |
|  | Two doses | -0.200 | [-0.229, -0.171] | 0.010 | [-0.136, 0.155] |
|  | Three doses | -0.232 | [-0.262, -0.203] | 0.596 | [0.542, 0.650] |
| Imported vaccine |  | -0.807 | [-0.846, -0.767] | 1.433 | [1.382, 1.484] |
| Number of observations | | 144,000.0 |  |  |  |
| Number of participants | | 12,000.0 |  |  |  |
| Waldχ2/LRχ2 |  | 113,475.6 |  |  |  |
| P>χ2 |  | <0.001 |  |  |  |
| Akaike Information Criterion (AIC) | | 202,972.9 |  |  |  |
| Bayesian Information Criterion (BIC) | | 203,210.0 |  |  |  |
| Log pseudo-likelihood | | -101,462.4 |  |  |  |

^a^ The signs of $\mu_{m}$ indicated whether the reference group preferred the vaccine attribute or not; positive coefficient meant this attribute was preferred and otherwise meant this attribute was not preferred. See Methods Section in the main text for further details of variable notations and model specifications.

^b^ The relationship of 95% CI of $\delta_{m}$ and 0 indicated whether there existed heterogeneity in this vaccine attribute level. If included, then no significant heterogeneity was found and if not, significant heterogeneity was found. Negative estimate of $\delta_{m}$ originates from the Apollo package where the sign of $\delta_{m}$ is not constrained for ease of coefficient estimation.^1^

Abbreviations: CI, confidence interval; SD, standard deviation; ASC, alternative-specific constant.

## References

1. ApolloChoice. FREQUENTLY ASKED QUESTIONS (FAQ) [Available from: <http://apollochoicemodelling.com/faq.html> accessed August 19 2023.
