# Supplementary material for: Preferences regarding COVID-19 vaccination among 12,000 adults in China: A cross-sectional discrete choice experiment
Source: PLOS Glob Public Health. 2024 Jul 11;4(7):e0003387. doi: 10.1371/journal.pgph.0003387 (PMC11239003; doi:10.1371/journal.pgph.0003387)
Supplement: S3 Table — (DOCX) [file pgph.0003387.s009.docx]

## S3 Table: Detailed results of the extended mixed logit model

**S3 Table** reports the coefficients of attribute levels among different subgroups from the extended mixed logit model. Results were presented for the total effect (main effect plus the interaction effect; $\nu_{m}+\gamma_{mk}$ in Equation (4) of **Method Section**) with confidence intervals adjusted accordingly, to identify the heterogeneity across the groups and present whether the subgroup population prefer the vaccine attribute or not.

Specifically, older adults were less sensitive than younger adults to price (coefficient -0.135, [-0.191, -0.079]), high risk (coefficient -0.992, [-1.214, -0.771], high doses (coefficient -0.022, [-0.184, 0.140]) and imported vaccine (coefficient: -0.389, [-0.624, -0.154]). In addition, the married population showed a similar pattern of heterogeneity as the older adults. They were less sensitive than those who are single to higher price (coefficient -0.175, [-0.225, -0.126]) and higher risk (coefficient -0.892, [-1.091, -0.694]).

In addition to the demographic characteristics, work status and income level also had significant impact on vaccine preferences. Unemployed participants were more averse to moderate risk (coefficient -1.090, [-1.249, -0.930]) and imported origin (coefficient -1.001, [-1.177, -0.825]) than employed participants. By contrast, higher-income participants attached greater importance to longer duration of protection (coefficient 1.522, [1.271, 1.773]) and high efficacy (coefficient 1.472, [1.249, 1.694]) than lower-income participants.

Chronic disease diagnosis history and vaccination history could also play important roles in vaccine preferences as well. Those participants ever diagnosed with a chronic disease reported less sensitive to long protection (coefficient 1.035, [0.739, 1.331]) and high efficacy (coefficient 0.798, [0.536, 1.060]). Those who had already received at least one vaccine dose exhibited greater preference for a vaccine offering long protection (coefficient 0.577, [0.396, 0.759]) and high efficacy (coefficient 1.305, [1.082, 1.527]) and were less sensitive to price (coefficient -0.166, [-0.216, -0.116]) and high risk (coefficient -0.912, [-1.109, -0.714])

**Table S3. Main effects plus interaction effects of attributes across groups**

| **Attribute** | **Attribute levels** | **Sex** | | | | | |
| --- | --- | --- | --- | --- | --- | --- | --- |
|  |  | **Male (Reference)** | | | **Female** | | |
|  |  | **Coefficient** | **95% CI** | | **Coefficient** | **95% CI** | |
| Price (per 100 RMB) |  | -0.224 | [-0.275, -0.173] | | -0.206* | [-0.257, -0.156] | |
| Risk of rare, but serious side-effects from the vaccine | No risk | Ref | - | | - | - | |
|  | 1/1,000,000 | -0.844 | [-1.031, -0.658] | | -0.868 | [-1.053, -0.683] | |
|  | 1/100,000 | -1.158 | [-1.359, -0.958] | | -1.181 | [-1.380, -0.981] | |
| Duration of protection | 6 months | Ref | - | | - | - | |
|  | 12 months | 0.393 | [0.209, 0.578] | | 0.447* | [0.264, 0.630] | |
|  | Life long | 1.272 | [1.016, 1.529] | | 1.408** | [1.154, 1.661] | |
| Degree of efficacy | 50% | Ref | - | | - | - | |
|  | 70% | 0.402 | [0.207, 0.598] | | 0.400 | [0.205, 0.596] | |
|  | 90% | 1.063 | [0.840, 1.287] | | 1.141* | [0.918, 1.364] | |
| Oral vaccine |  | -0.211 | [-0.377, -0.046] | | -0.209 | [-0.374, -0.043] | |
| Frequency of vaccination | 1 dose | Ref | - | | - | - | |
|  | 2 doses | -0.205 | [-0.233, -0.176] | | -0.205 | [-0.233, -0.176] | |
|  | 3 doses | -0.148 | [-0.296, 0.000] | | -0.129 | [-0.278, 0.019] | |
| Imported vaccine |  | -0.653 | [-0.864, -0.443] | | -0.658 | [-0.867, -0.449] | |
| **Attribute** | **Attribute levels** | **Age Group** | | | | | |
|  |  | **18-39 years (Reference)** | | **40-59 years** | | **60 years and above** | |
|  |  | **Coefficient** | **95% CI** | **Coefficient** | **95% CI** | **Coefficient** | **95% CI** |
| Price (per 100 RMB) |  | -0.224 | [-0.275, -0.173] | -0.214 | [-0.272, -0.157] | -0.135*** | [-0.191, -0.079] |
| Risk of rare, but serious side-effects from the vaccine | No risk | Ref | - | - | - | - | - |
|  | Moderate risk | -0.844 | [-1.031, -0.658] | -0.981** | [-1.193, -0.769] | -0.707* | [-0.915, -0.499] |
|  | High risk | -1.158 | [-1.359, -0.958] | -1.194 | [-1.420, -0.968] | -0.992** | [-1.214, -0.771] |
| Duration of protection | 6 months | Ref | - | - | - | - | - |
|  | 12 months | 0.393 | [0.209, 0.578] | 0.451 | [0.241, 0.660] | 0.493 | [0.288, 0.698] |
|  | Life long | 1.272 | [1.016, 1.529] | 1.269 | [0.983, 1.555] | 1.224 | [0.943, 1.505] |
| Degree of efficacy | 50% | Ref | - | - | - | - | - |
|  | 70% | 0.402 | [0.207, 0.598] | 0.437 | [0.212, 0.661] | 0.481 | [0.261, 0.702] |
|  | 90% | 1.063 | [0.840, 1.287] | 1.112 | [0.859, 1.365] | 1.081 | [0.832, 1.331] |
| Oral vaccine |  | -0.211 | [-0.377, -0.046] | -0.227 | [-0.415, -0.039] | -0.176 | [-0.360, 0.007] |
| Frequency of vaccination | 1 dose | Ref | - | - | - | - | - |
|  | 2 doses | -0.205 | [-0.233, -0.176] | -0.205 | [-0.233, -0.176] | -0.205 | [-0.233, -0.176] |
|  | 3 doses | -0.148 | [-0.296, 0.000] | -0.113 | [-0.279, 0.054] | -0.022** | [-0.184, 0.140] |
| Imported vaccine |  | -0.653 | [-0.864, -0.443] | -0.511* | [-0.749, -0.272] | -0.389*** | [-0.624, -0.154] |
| **Attribute** | **Attribute levels** | **Education** | | | | | |
|  |  | **Primary school or less (Reference)** | | **Middle and high school** | | **College and above** | |
|  |  | **Coefficient** | **95% CI** | **Coefficient** | **95% CI** | **Coefficient** | **95% CI** |
| Price (per 100 RMB) |  | -0.224 | [-0.275, -0.173] | -0.221 | [-0.271, -0.171] | -0.312*** | [-0.370, -0.253] |
| Risk of rare, but serious side-effects from the vaccine | No risk | Ref | - | - | - | - | - |
|  | Moderate risk | -0.844 | [-1.031, -0.658] | -0.750 | [-0.933, -0.567] | -0.877 | [-1.088, -0.667] |
|  | High risk | -1.158 | [-1.359, -0.958] | -1.079 | [-1.277, -0.881] | -1.19 | [-1.416, -0.965] |
| Duration of protection | 6 months | Ref | - | - | - | - | - |
|  | 12 months | 0.393 | [0.209, 0.578] | 0.407 | [0.223, 0.591] | 0.310 | [0.102, 0.519] |
|  | Life long | 1.272 | [1.016, 1.529] | 1.361 | [1.105, 1.618] | 1.348 | [1.055, 1.641] |
| Degree of efficacy | 50% | Ref | - | - | - | - | - |
|  | 70% | 0.402 | [0.207, 0.598] | 0.476 | [0.279, 0.672] | 0.361 | [0.134, 0.587] |
|  | 90% | 1.063 | [0.840, 1.287] | 1.125 | [0.903, 1.347] | 1.050 | [0.792, 1.308] |
| Oral vaccine |  | -0.211 | [-0.377, -0.046] | -0.190 | [-0.356, -0.025] | -0.226 | [-0.416, -0.036] |
| Frequency of vaccination | 1 dose | Ref | - | - | - | - | - |
|  | 2 doses | -0.205 | [-0.233, -0.176] | -0.205 | [-0.233, -0.176] | -0.205 | [-0.233, -0.176] |
|  | 3 doses | -0.148 | [-0.296, 0.000] | -0.184 | [-0.330, -0.039] | -0.269* | [-0.437, -0.102] |
| Imported vaccine |  | -0.653 | [-0.864, -0.443] | -0.749 | [-0.961, -0.538] | -0.941*** | [-1.186, -0.697] |
| **Attribute** | **Attribute levels** | **Marriage** | | | | | |
|  |  | **Married** | | | **Not in a marriage (Reference)** | | |
|  |  | **Coefficient** | **95% CI** | | **Coefficient** | **95% CI** | |
| Price (per 100 RMB) |  | -0.175*** | [-0.225, -0.126] | | -0.224 | [-0.275, -0.173] | |
| Risk of rare, but serious side-effects from the vaccine | No risk | - | - | | Ref | - | |
|  | Moderate risk | -0.674*** | [-0.858, -0.489] | | -0.844 | [-1.031, -0.658] | |
|  | High risk | -0.892*** | [-1.091, -0.694] | | -1.158 | [-1.359, -0.958] | |
| Duration of protection | 6 months | - | - | | Ref | - | |
|  | 12 months | 0.397 | [0.216, 0.579] | | 0.393 | [0.209, 0.578] | |
|  | Life long | 1.217 | [0.965, 1.469] | | 1.272 | [1.016, 1.529] | |
| Degree of efficacy | 50% | - | - | | Ref | - | |
|  | 70% | 0.389 | [0.196, 0.583] | | 0.402 | [0.207, 0.598] | |
|  | 90% | 0.998 | [0.776, 1.221] | | 1.063 | [0.840, 1.287] | |
| Oral vaccine |  | -0.181 | [-0.345, -0.017] | | -0.211 | [-0.377, -0.046] | |
| Frequency of vaccination | 1 dose | - | - | | Ref | - | |
|  | 2 doses | -0.205 | [-0.233, -0.176] | | -0.205 | [-0.233, -0.176] | |
|  | 3 doses | -0.168 | [-0.315, -0.021] | | -0.148 | [-0.296, 0.000] | |
| Imported vaccine |  | -0.652 | [-0.859, -0.445] | | -0.653 | [-0.864, -0.443] | |
| **Attribute** | **Attribute levels** | **Residence** | | | | | |
|  |  | **Rural (Reference)** | | | **Urban** | | |
|  |  | **Coefficient** | **95% CI** | | **Coefficient** | **95% CI** | |
| Price (per 100 RMB) |  | -0.224 | [-0.275, -0.173] | | -0.226 | [-0.278, -0.175] | |
| Risk of rare, but serious side-effects from the vaccine | No risk | Ref | - | | - | - | |
|  | Moderate risk | -0.844 | [-1.031, -0.658] | | -0.901 | [-1.093, -0.710] | |
|  | High risk | -1.158 | [-1.359, -0.958] | | -1.187 | [-1.392, -0.983] | |
| Duration of protection | 6 months | Ref | - | | - | - | |
|  | 12 months | 0.393 | [0.209, 0.578] | | 0.411 | [0.222, 0.599] | |
|  | Life long | 1.272 | [1.016, 1.529] | | 1.371* | [1.107, 1.634] | |
| Degree of efficacy | 50% | Ref | - | | - | - | |
|  | 70% | 0.402 | [0.207, 0.598] | | 0.433 | [0.231, 0.634] | |
|  | 90% | 1.063 | [0.840, 1.287] | | 1.065 | [0.835, 1.295] | |
| Oral vaccine |  | -0.211 | [-0.377, -0.046] | | -0.196 | [-0.366, -0.026] | |
| Frequency of vaccination | 1 dose | Ref | - | | - | - | |
|  | 2 doses | -0.205 | [-0.233, -0.176] | | -0.205 | [-0.233, -0.176] | |
|  | 3 doses | -0.148 | [-0.296, 0.000] | | -0.135 | [-0.286, 0.017] | |
| Imported vaccine |  | -0.653 | [-0.864, -0.443] | | -0.752** | [-0.967, -0.537] | |
| **Attribute** | **Attribute levels** | **Work situation** | | | | | |
|  |  | **Work in a private setting (Reference)** | | **Work in a public setting** | | **No job** | |
|  |  | **Coefficient** | **95% CI** | **Coefficient** | **95% CI** | **Coefficient** | **95% CI** |
| Price (per 100 RMB) |  | -0.224 | [-0.275, -0.173] | -0.210 | [-0.262, -0.159] | -0.243 | [-0.286, -0.200] |
| Risk of rare, but serious side-effects from the vaccine | No risk | Ref | - | - | - | - | - |
|  | Moderate risk | -0.844 | [-1.031, -0.658] | -0.891 | [-1.078, -0.703] | -1.090*** | [-1.249, -0.930] |
|  | High risk | -1.158 | [-1.359, -0.958] | -1.187 | [-1.388, -0.985] | -1.227 | [-1.395, -1.059] |
| Duration of protection | 6 months | Ref | - | - | - | - | - |
|  | 12 months | 0.393 | [0.209, 0.578] | 0.416 | [0.232, 0.600] | 0.373 | [0.220, 0.525] |
|  | Life long | 1.272 | [1.016, 1.529] | 1.333 | [1.076, 1.591] | 1.458* | [1.247, 1.669] |
| Degree of efficacy | 50% | Ref | - | - | - | - | - |
|  | 70% | 0.402 | [0.207, 0.598] | 0.508** | [0.312, 0.703] | 0.467 | [0.302, 0.631] |
|  | 90% | 1.063 | [0.840, 1.287] | 1.169* | [0.944, 1.394] | 1.227* | [1.040, 1.414] |
| Oral vaccine |  | -0.211 | [-0.377, -0.046] | -0.194 | [-0.360, -0.028] | -0.201 | [-0.339, -0.063] |
| Frequency of vaccination | 1 dose | Ref | - | - | - | - | - |
|  | 2 doses | -0.205 | [-0.233, -0.176] | -0.205 | [-0.233, -0.176] | -0.205 | [-0.233, -0.176] |
|  | 3 doses | -0.148 | [-0.296, 0.000] | -0.160 | [-0.309, -0.012] | -0.263* | [-0.387, -0.138] |
| Imported vaccine |  | -0.653 | [-0.864, -0.443] | -0.691 | [-0.903, -0.478] | -1.001*** | [-1.177, -0.825] |
| **Attribute** | **Attribute levels** | **Insurance type** | | | | | |
|  |  | **BMIURR (Reference)** | | | **UEBMI** | | |
|  |  | **Coefficient** | **95% CI** | | **Coefficient** | **95% CI** | |
| Price (per 100 RMB) |  | -0.224 | [-0.275, -0.173] | | -0.272** | [-0.316, -0.229] | |
| Risk of rare, but serious side-effects from the vaccine | No risk | Ref | - | | - | - | |
|  | Moderate risk | -0.844 | [-1.031, -0.658] | | -0.911 | [-1.074, -0.748] | |
|  | High risk | -1.158 | [-1.359, -0.958] | | -1.165 | [-1.338, -0.992] | |
| Duration of protection | 6 months | Ref | - | | - | - | |
|  | 12 months | 0.393 | [0.209, 0.578] | | 0.273* | [0.118, 0.428] | |
|  | Life long | 1.272 | [1.016, 1.529] | | 1.241 | [1.026, 1.456] | |
| Degree of efficacy | 50% | Ref | - | | - | - | |
|  | 70% | 0.402 | [0.207, 0.598] | | 0.301 | [0.134, 0.467] | |
|  | 90% | 1.063 | [0.840, 1.287] | | 0.944 | [0.758, 1.131] | |
| Oral vaccine |  | -0.211 | [-0.377, -0.046] | | -0.191 | [-0.332, -0.051] | |
| Frequency of vaccination | 1 dose | Ref | - | | - | - | |
|  | 2 doses | -0.205 | [-0.233, -0.176] | | -0.205 | [-0.233, -0.176] | |
|  | 3 doses | -0.148 | [-0.296, 0.000] | | -0.223 | [-0.348, -0.098] | |
| Imported vaccine |  | -0.653 | [-0.864, -0.443] | | -0.727 | [-0.905, -0.549] | |
| **Attribute** | **Attribute levels** | **Income level** | | | | | |
|  |  | **< 60,000 RMB (Reference)** | | **60,000 - 149,999 RMB** | | **≥ 150,000 RMB** | |
|  |  | **Coefficient** | **95% CI** | **Coefficient** | **95% CI** | **Coefficient** | **95% CI** |
| Price (per 100 RMB) |  | -0.224 | [-0.275, -0.173] | -0.211 | [-0.260, -0.163] | -0.204 | [-0.254, -0.153] |
| Risk of rare, but serious side-effects from the vaccine | No risk | Ref | - | - | - | - | - |
|  | Moderate risk | -0.844 | [-1.031, -0.658] | -0.868 | [-1.044, -0.692] | -0.940* | [-1.124, -0.755] |
|  | High risk | -1.158 | [-1.359, -0.958] | -1.123 | [-1.312, -0.934] | -1.150 | [-1.345, -0.955] |
| Duration of protection | 6 months | Ref | - | - | - | - | - |
|  | 12 months | 0.393 | [0.209, 0.578] | 0.382 | [0.210, 0.554] | 0.379 | [0.198, 0.560] |
|  | Life long | 1.272 | [1.016, 1.529] | 1.332 | [1.094, 1.570] | 1.522*** | [1.271, 1.773] |
| Degree of efficacy | 50% | Ref | - | - | - | - | - |
|  | 70% | 0.402 | [0.207, 0.598] | 0.447 | [0.263, 0.631] | 0.619*** | [0.425, 0.814] |
|  | 90% | 1.063 | [0.840, 1.287] | 1.255*** | [1.043, 1.467] | 1.472*** | [1.249, 1.694] |
| Oral vaccine |  | -0.211 | [-0.377, -0.046] | -0.208 | [-0.364, -0.051] | -0.148 | [-0.310, 0.014] |
| Frequency of vaccination | 1 dose | Ref | - | - | - | - | - |
|  | 2 doses | -0.205 | [-0.233, -0.176] | -0.205 | [-0.233, -0.176] | -0.205 | [-0.233, -0.176] |
|  | 3 doses | -0.148 | [-0.296, 0.000] | -0.157 | [-0.297, -0.017] | -0.195 | [-0.339, -0.050] |
| Imported vaccine |  | -0.653 | [-0.864, -0.443] | -0.510** | [-0.705, -0.315] | -0.613 | [-0.817, -0.409] |
| **Attribute** | **Attribute levels** | **Chronic conditions** | | | | | |
|  |  | **Ever diagnosed with a chronic disease** | | | **Not ever (Reference)** | | |
|  |  | **Coefficient** | **95% CI** | | **Coefficient** | **95% CI** | |
| Price (per 100 RMB) |  | -0.235 | [-0.294, -0.176] | | -0.224 | [-0.275, -0.173] | |
| Risk of rare, but serious side-effects from the vaccine | No risk | - | - | | Ref | - | |
|  | Moderate risk | -0.744 | [-0.961, -0.528] | | -0.844 | [-1.031, -0.658] | |
|  | High risk | -1.064 | [-1.299, -0.829] | | -1.158 | [-1.359, -0.958] | |
| Duration of protection | 6 months | - | - | | Ref | - | |
|  | 12 months | 0.339 | [0.128, 0.550] | | 0.393 | [0.209, 0.578] | |
|  | Life long | 1.035** | [0.739, 1.331] | | 1.272 | [1.016, 1.529] | |
| Degree of efficacy | 50% | - | - | | Ref | - | |
|  | 70% | 0.296 | [0.065, 0.526] | | 0.402 | [0.207, 0.598] | |
|  | 90% | 0.798*** | [0.536, 1.060] | | 1.063 | [0.840, 1.287] | |
| Oral vaccine |  | -0.230 | [-0.421, -0.038] | | -0.211 | [-0.377, -0.046] | |
| Frequency of vaccination | 1 dose | - | - | | Ref | - | |
|  | 2 doses | -0.205 | [-0.233, -0.176] | | -0.205 | [-0.233, -0.176] | |
|  | 3 doses | -0.101 | [-0.270, 0.069] | | -0.148 | [-0.296, 0.000] | |
| Imported vaccine |  | -0.558 | [-0.801, -0.314] | | -0.653 | [-0.864, -0.443] | |
| **Attribute** | **Attribute levels** | **COVID-19 vaccination history** | | | | | |
|  |  | **Vaccinated at least one dose** | | | **Not yet (Reference)** | | |
|  |  | **Coefficient** | **95% CI** | | **Coefficient** | **95% CI** | |
| Price (per 100 RMB) |  | -0.166*** | [-0.216, -0.116] | | -0.224 | [-0.275, -0.173] | |
| Risk of rare, but serious side-effects from the vaccine | No risk | - | - | | Ref | - | |
|  | Moderate risk | -0.583*** | [-0.766, -0.399] | | -0.844 | [-1.031, -0.658] | |
|  | High risk | -0.912*** | [-1.109, -0.714] | | -1.158 | [-1.359, -0.958] | |
| Duration of protection | 6 months | - | - | | Ref | - | |
|  | 12 months | 0.577*** | [0.396, 0.759] | | 0.393 | [0.209, 0.578] | |
|  | Life long | 1.459*** | [1.206, 1.712] | | 1.272 | [1.016, 1.529] | |
| Degree of efficacy | 50% | - | - | | Ref | - | |
|  | 70% | 0.611*** | [0.418, 0.805] | | 0.402 | [0.207, 0.598] | |
|  | 90% | 1.305*** | [1.082, 1.527] | | 1.063 | [0.840, 1.287] | |
| Oral vaccine |  | -0.197 | [-0.362, -0.032] | | -0.211 | [-0.377, -0.046] | |
| Frequency of vaccination | 1 dose | - | - | | Ref | - | |
|  | 2 doses | -0.205 | [-0.233, -0.176] | | -0.205 | [-0.233, -0.176] | |
|  | 3 doses | -0.119 | [-0.266, 0.029] | | -0.148 | [-0.296, 0.000] | |
| Imported vaccine |  | -0.705 | [-0.916, -0.495] | | -0.653 | [-0.864, -0.443] | |

^a^ The signs of main effects plus interaction effects $\nu_{m}+\gamma_{mk}$, see Methods Section in the main text for further details of variable notations and model specifications.) indicated that whether the group preferred the vaccine attribute or not; positive effect meant this attribute was preferred and otherwise meant this attribute was not preferred. The asterisks indicated the significance level of interaction term $\gamma_{mk}$: *** p value < 0.001; ** p value < 0.01; * p value < 0.05. Asterisks meant that there was preference heterogeneity found between the specific group and reference group.

^b^ Since the interactions between two doses and participants’ characteristics are excluded in the extended mixed logit model, the total effects of two doses were the same across different groups.
