# Supplementary material for: Preferences regarding COVID-19 vaccination among 12,000 adults in China: A cross-sectional discrete choice experiment
Source: PLOS Glob Public Health. 2024 Jul 11;4(7):e0003387. doi: 10.1371/journal.pgph.0003387 (PMC11239003; doi:10.1371/journal.pgph.0003387)
Supplement: Attachment — Submitted filename: Response to reviewers_0503.docx [file pgph.0003387.s010.docx]

May 03, 2024

Dr. Catherine Kyobutungi and Professor Madhukar Pai

Editors-in-Chief

*PLOS Global Public Health*

**Resubmission of manuscript** PGPH-D-23-02386**:** Preferences regarding COVID-19 vaccination among 12,000 adults in China: a cross-sectional discrete choice experiment

Dear Dr. Kyobutungi and Professor Pai:

Thank you very much for your positive evaluation of our paper and for providing us with important and constructive comments on behalf of the editors and reviewers. We would also like to thank the editorial committee and the two reviewers for their valuable and insightful comments and suggestions. We have carefully addressed each comment and have incorporated the suggested changes.

Below, we describe in detail how we have taken the journal requirements and reviewers’ comments and suggestions into account in our revised manuscript. We have also carefully edited the entire manuscript and the appendices to ensure the coherence and clarity of our revised text.

Sincerely,

Fengyun Yu, Lirui Jiao, Qiushi Chen, Qun Wang, Manuela De Allegri, Zhong Cao, Wenjin Chen, Xuedi Ma, Chao Wang, Jonas Wachinger, Zhangfeng Jin, Aditi Bunker, Pascal Geldsetzer, Juntao Yang, Lan Xue, Till Bärnighausen, Simiao Chen

Journal Requirements

We thank the editors for the valuable feedback in helping us adhere to the journal requirements. We have carefully addressed each point.

Below, we explain in detail how we have addressed each of the comments. We show the comments in italics and our responses in standard font.

*1. Please include a complete copy of PLOS’ questionnaire on inclusivity in global research in your revised manuscript. Our policy for research in this area aims to improve transparency in the reporting of research performed outside of researchers’ own country or community. The policy applies to researchers who have travelled to a different country to conduct research, research with Indigenous populations or their lands, and research on cultural artefacts. The questionnaire can also be requested at the journal’s discretion for any other submissions, even if these conditions are not met.  Please find more information on the policy and a link to download a blank copy of the questionnaire here: https://journals.plos.org/globalpublichealth/s/best-practices-in-research-reporting. Please upload a completed version of your questionnaire as Supporting Information when you resubmit your manuscript.*

Thank you very much for your helpful comments. We have attached the PLOS’ questionnaire in our resubmission.

*2. Please amend your detailed Financial Disclosure statement. This is published with the article. It must therefore be completed in full sentences and contain the exact wording you wish to be published.*

*a. State the initials, alongside each funding source, of each author to receive each grant.
b. State what role the funders took in the study. If the funders had no role in your study, please state: “The funders had no role in study design, data collection and analysis, decision to publish, or preparation of the manuscript.”
c. If any authors received a salary from any of your funders, please state which authors and which funders.*

*If you did not receive any funding for this study, please simply state: “The authors received no specific funding for this work.*

Thank you very much for your comment. We have amended the following financial disclosure statement:

“This work was supported by the Chinesisch-Deutsches Zentrum für Wissenschaftsförderung under grant number C0048 to SC. The funders had no role in study design, data collection and analysis, decision to publish, or preparation of the manuscript.”

3. *Please provide separate figure files in .tif or .eps format only and remove any figures embedded in your manuscript file. Please also ensure all files are under our size limit of 10MB.*

*For more information about figure files please see our guidelines:*

*https://journals.plos.org/globalpublichealth/s/figures* 
[*https://journals.plos.org/globalpublichealth/s/figures#loc-file-requirement*](https://journals.plos.org/globalpublichealth/s/figures#loc-file-requirement)

Thank you very much for pointing this out. We have provided all separate figure files in “.eps” format following the guidelines and removed those that were previously embedded in the manuscript.

4. *Some material included in your submission may be copyrighted. According to PLOS’s copyright policy, authors who use figures or other material (e.g., graphics, clipart, maps) from another author or copyright holder must demonstrate or obtain permission to publish this material under the Creative Commons Attribution 4.0 International (CC BY 4.0) License used by PLOS journals. Please closely review the details of PLOS’s copyright requirements here: PLOS Licenses and Copyright. If you need to request permissions from a copyright holder, you may use PLOS's Copyright Content Permission form.*

*Please respond directly to this email or email the journal office and provide any known details concerning your material's license terms and permissions required for reuse, even if you have not yet obtained copyright permissions or are unsure of your material's copyright compatibility.*

*Potential Copyright Issues:*

*Figs 4: please (a) provide a direct link to the base layer of the map (i.e., the country or region border shape) and ensure this is also included in the figure legend; and (b) provide a link to the terms of use / license information for the base layer image or shapefile. We cannot publish proprietary or copyrighted maps (e.g. Google Maps, Mapquest) and the terms of use for your map base layer must be compatible with our CC-BY 4.0 license.*

Thank you very much for this suggestion. The map data in “.json” format is obtained from the Ministry of Civil Affairs of the People’s Republic of China (URL: [xzqh.mca.gov.cn/data/quanguo.json](http://xzqh.mca.gov.cn/data/quanguo.json)), which provides the administrative divisions of China accessible to everyone. As per requirements, we have also included the link in the figure legend.

Furthermore, the copyright information for the layer image is available on the site dmfw.mca.gov.cn/version.html. This statement is generally CC-BY 4.0 compliant in terms of attribution and non-commercial use.

Responses to Reviewers

**Responses to Reviewer 1**

*I thank the authors for conducting a very thorough study to explore the impact of vaccine attributes and other determinants on vaccine uptake. To my knowledge, no other study that has looked at so many different attributes influencing COVID-19 vaccine uptake.

This study, using the discrete choice experiment, determined various vaccine related attributes that influence vaccine uptake. The authors looked at many different attributes, including vaccine efficacy, dose requirement, country of production and potential side effects. They also explored individual level determinants that are associated with vaccine uptake such as age, education level, work situation, income level, etc. Moreover, the authors looked at vaccine preferences among populations living in different geographical locations in China. And finally, they predicted vaccine uptake probability of a hypothetical vaccine to further demonstrate the geographical heterogeneities based on pricing in vaccine uptake. I agree with the authors that these findings would help in designing targeted vaccine campaigns for vaccines, especially adult vaccines, in the future.
The authors of this study have tried to create a nationally representative sample while acknowledging the limitations of selection bias.

Overall, the study is well written. However, I would request the authors to re-assess their discussion section to make it more thorough and ensure all the major findings are discussed (especially the points that are mentioned in the abstract).*

We thank Reviewer 1 for the valuable and constructive comments. We have carefully incorporated the reviewer’s advice, and our paper has improved substantially as a result.

Below, we explain in detail how we have taken each of the comments into account in our revised manuscript. We show the reviewer’s comments in italics and our responses in standard font.

*1. Introduction: Please mention the date (month and year) instead of “At the time of writing,..”. With the ever-changing situation related to COVID-19, it will allow the reader to get a general idea of what the situation of the pandemic was when this study was written.*

Thank you very much for your constructive feedback. We modified the original introduction to mention the accurate date as following (**Page 5, Line 79-82**):

“In February 2023, thirteen COVID-19 vaccines have been approved for emergency use by World Health Organization (WHO), including inactivated vaccines, viral vector vaccines, mRNA vaccines, and protein subunit vaccines.^1^”

*2. Discussion: Line 398-402: The authors compare their findings of vaccine uptake preferences in July 2021, after vaccines were available and had been administered for over six-months (China began vaccinating in December 2020), with studies from other countries (France and Chile) that predicted vaccine uptake when vaccines were not available. For a fair comparison, please share stats from studies from China, if any, that were conducted before the vaccines were available. Trust in the government, communication strategies, etc. can be very different in different countries.*

Thank you very much for your insightful comments. Following your suggestion, we found an earlier study in China that were conducted before the vaccines were available and have revised our discussion accordingly as follows (**Page 23, Line 407-411**):

“Our findings show a sharp increase in the percentage of people who hold positive preferences toward vaccination, compared with the findings from an earlier study in China prior to vaccine availability that showed 80% of Chinese respondents would be willing to take the vaccine when it becomes available.^2^”

*3. Line 431-450: The authors suggest that health communication efforts in China could have had a positive impact on people’s willingness to pay for the vaccine. For some people, that might be the case. However, for most of the population, the ability to pay for the vaccine, especially when the cost is as steep as 1,000 RMB would be the deciding factor. Therefore, the authors should further elaborate on what could be the reason for this finding. Cost of the vaccine is a big barrier when it comes to predicting vaccine uptake. Is it possible that the survey participants believed that whatever the price of the vaccine, they will not have to pay for it out of pocket and it will be covered by their insurance? How does the health insurance work in China? Would insurance have covered the cost of the vaccine for those who had health insurance?* *Interestingly, in this study, adults with high income were not significantly sensitive to the price of the vaccine. (Figure 1 a)*

Thank you very much for your insightful comments. We agree that it is possible that the survey participants believed that they would not have to pay for the COVID-19 vaccines out-of-pocket, likely not because the vaccines would be covered by insurance, but rather due to the government’s earlier announcement that COVID-19 vaccination would be free to all citizens. In addition, health insurance in China in general has not covered influenza vaccines. We have added the following content to elaborate on cost as a reason for the willingness to pay (WTP) for COVID-19 vaccines in our findings (**Page 25, Line 455-473**):

“Another possible explanation for the increase in WTP from 600 RMB in September 2020 to 1000 RMB in 2021 may be that by the time the participants were surveyed in this study, the Chinese government had already announced that COVID-19 vaccinations would be offered free to Chinese citizens^3^ and so participants may have believed that they would not have to pay for COVID-19 vaccines out-of-pocket regardless of vaccine price. Knowing that vaccines are provided for free might change the behavior of participants through cognitive mechanisms such as anchoring effects, which is the tendency for participants to rely heavily on the first piece of information when making decisions.^4^ When vaccines are announced as free, this sets an initial anchor of zero cost, which may lead participants to express a high WTP as a form of positive feedback or approval of the free vaccine initiative, even if they do not actually expect to pay that amount – this may also explain our findings of the lower sensitivity to vaccine price among adults with high income. However, not all participants may have been aware of the free COVID-19 vaccination announcement and there could have been uncertainty about how long the free COVID-19 vaccination may last for. Furthermore, this anchoring effect may be moderated by the fact that influenza vaccines in the past were typically paid out-of-pocket and were only partially or fully subsidized for children and older adults via health insurance or local government funds in economically developed regions in China.^5 6^”

*4. Lines 452-460: Vaccine uptake probability across different provinces was high (76.5% to 89.7%) when the price was 0 RMB, which reduced to 49.8% to 71.5% when the price was increased to 600 RMB. (line 373-376) However, the authors quote that a contributing factor to this drop could be the different composition of occupational groups in different states. This does not appear to be a plausible explanation unless a significant proportion of population is different based on occupation. Could there be other reasons such as differences on the bases of urban and rural, education, affordability, income etc. It’s not clear that if price changes, why would the healthcare workers suddenly be less willing to be vaccinated in Shanghai.*

Thank you very much for your keen insights. We agree that different composition of occupational groups in different states may not be a plausible explanation since it is difficult to capture a significant proportion of the population that are different based on occupation across geographical regions. As you suggested, we think that living affordability could be an important contributing factor to the steep drop in vaccine uptake probability in coastal regions. We have thus removed the discussion on healthcare workers and revised the previous Lines 452-460 to improve clarity and logic as following (**Page 26, Line 483-485**):

“This could be attributed to the higher sensitivity to price changes in coastal regions as a result of higher living costs despite potentially higher average incomes.^7^”

*5. Can the authors also elaborate on why the health care workers are less willing to get vaccinated?*

Thank you very much for your helpful comment. Following your previous suggestion, we revised our explanation for the geographical heterogeneities in vaccine uptake in our findings and removed the “different composition of occupational groups” argument, which involved discussing the low willingness to get vaccinated among healthcare workers. While this information is no longer in the manuscript, we completely agree that understanding why healthcare workers are less willing to get vaccinated is important to keep in mind. Vaccine hesitancy in healthcare workers have existed prior to COVID-19. In fact, studies have found that as low as 31% of healthcare workers were willing to receive the H1N1 vaccine. Existing literature show that barriers to COVID-19 vaccination in healthcare workers include concerns about vaccine safety, efficacy, speed of development, mistrust in state/public health response to COVID-19, and more.^8^ In particular, a study that examines COVID-19 vaccine acceptance among healthcare workers in China identified concerns about vaccine safety as the main reason for vaccine hesitancy.^9^ However, it is not clear why healthcare workers may have higher vaccine hesitancy than others in some cases, and there have been mixed findings with regards to this, with some studies finding that healthcare workers are more willing to be vaccinated than non-healthcare workers and other studies finding that vaccine uptake in healthcare workers is generally low.^10 11^

*6. Line 462-470: The study found that education was inversely associated with vaccine uptake. This is contradictory to findings from other studies (Viswanath et al. BMC Public Health (2021) 21:818 https://doi.org/10.1186/s12889-021-10862-1) that found education to be positively associated with vaccine uptake. Moreover, according to the world bank (https://www.worldbank.org/en/country/china/overview#:~:text=China%20is%20now%20an%20upper,in%20upper%2Dmiddle%20income%20countries), China is now an upper-middle-income country. So, comparing the findings of this study with LMICs is not justified.*

Thank you very much for your constructive comment. The reason why our finding that education was inversely associated with vaccine uptake is contradictory to findings from Viswanath et al. and other studies may be due to different settings and different levels of health education across countries. Viswanath et al. present a US setting where health education may be more closely related to the general education level than in China. Following the reviewer’s suggestions, we have removed the comparison in the findings from this study with those from LMICs and made revisions accordingly as follows (**Page 26-27, Line 497-502**):

“However, it is important to note that education level is found to be positively associated with vaccine uptake in most countries and regions.^12 13^ The discrepancy in findings may be attributed to the varying level of health education or vaccine-related education in relation to general education level in different countries (i.e., if higher general education level necessarily leads to higher awareness and knowledge of the benefits of vaccination).”

*7.* *Misinformation has been a huge menace to the COVID-19 pandemic. Study by Dhawan et al., (Dhawan et al. (2021) COVID-19 News and Misinformation: Do They Matter for Public Health Prevention?, Journal of Health Communication, 26:11, 799-808, DOI: 10.1080/10810730.2021.2010841) found* *that people who endorse more misinformation are less likely to follow behaviors to prevent COVID-19. Since the authors mention misinformation as one of the plausible reasons behind low vaccine uptake among people with higher education, can the authors also talk about the issue of misinformation in China? It will be of interest to the readers to learn about the spread of misinformation in China affecting vaccine uptake despite having a more regulated media environment.*

Thank you very much for your great suggestion. Informal sources such as social media have contributed heavily to misinformation regarding COVID-19 vaccination in China. We have cited the excellent study by Dhawan et al. and added the following component (**Page 26, Line 493-496**):

“People who endorse more misinformation are less likely to follow behaviors to prevent COVID-19.^14^ Specifically, social media usage was identified as the main source of COVID-19 vaccine misinformation in China, and public health authorities and professionals should make an active effort to debunk false claims.^15^”

*8. The authors can refer to studies below to provide recommendations for targeted vaccine campaigns to promote vaccine uptake based on the findings of this study:*

*a. Viswanath et al. (2021) Individual and social determinants of COVID-19 vaccine uptake. BMC Public Health 21, 818. https://doi.org/10.1186/s12889-021-10862-1*

*b. Dhawan et al. (2021) COVID-19 News and Misinformation: Do They Matter for Public Health Prevention?, Journal of Health Communication, 26:11, 799-808, DOI: 10.1080/10810730.2021.2010841*

*c. Bekalu et al. (2021) Adherence to COVID-19 mitigation measures among American adults: the need for consistent and unified messaging, Health Education Research, Volume 36, Issue 2, April 2021, Pages 178–191, https://doi.org/10.1093/her/cyab002*

Thank you very much for providing these excellent references. The three studies that the reviewer recommended all emphasize the need for effective public health messaging in combating COVID-19. This is highly relevant for our study findings, because better public health communication about the vaccines would address misinformation and improve vaccine uptake. We have added these references to the manuscript as follows (**Page 27-28, Line 519-521**):

“In particular, sending consistent and unified vaccine-related messages on media is much needed to facilitate trust and combat misinformation.^12 14 16^”

*9. The study establishes pricing-dependent geographical heterogeneities in vaccine uptake. However, the authors do not sufficiently establish the potential reasons for these differences nor suggest any recommendations for policy makers.*

Thank you very much for your constructive feedback. We have added the following to better establish the potential reason for pricing-dependent geographical heterogeneities in vaccine uptake (**Page 26, Line 483-485**):

“This could be attributed to the higher sensitivity to price changes in coastal regions as a result of higher living costs despite potentially higher average incomes.^7^”

In addition, we have added the following recommendation for policy makers accordingly (**Page 27, Line 511-513**):

“Furthermore, targeted vaccine campaigns in specific geographic regions can be helpful in promoting vaccine uptake, especially in regions that are more price-sensitive.”

Responses to Reviewers

**Responses to Reviewer 2**

*This study adopted a discrete choice experiment to elicit public preference on COVID-19 vaccination among 12,000 adults. It is one of the few studies using a nationwide sample to answer the questions on COVID-19 preference. Despite this, there are a few details in the study requiring clarifications.*

We appreciate the reviewer’s positive feedback on the overall study. We are grateful for the reviewer’s constructive comments on our experiment design and sampling, which have helped us further improve the quality of our paper. In the following, we provide point-by-point responses to each of the reviewer’s specific comments.

*1. Why include cost as one of the attributes? I believe at the time of the survey, June-July 2021, the COVID-19 vaccination programme has been launched in China free of charge from the public. People do not have to consider cost of vaccination when making their decisions. Adding this attribute does not match to the real-world scenario.*

Thank you very much for your insightful comments. We agree that COVID-19 vaccines were distributed free of charge at the time of our survey and one study excluded vaccine cost for this reason.^17^ Though individuals do not bear the cost of the vaccination, we still included the cost as one of the attributes out of the following reasons:

1) Preliminary evidence: In our preliminary literature review and qualitative online interviews, cost is always the crucial factor influencing respondents’ choices.^2 18-21^ Cost remains a critical factor influencing vaccine acceptance, even when it’s not directly borne by individuals. People still perceive value in terms of cost-effectiveness.
2) Willingness to pay: By including cost, we can assess individuals’ willingness to pay (WTP) for the vaccine. WTP provides insights into how individuals value different vaccine attributes and underscores the significance of addressing issues of vaccines related to affordability.^22^ As you mentioned, we finally chose vaccine uptake probability to measure the significance of vaccine attributes due to the consideration of real-world scenarios, but it doesn’t imply that we should exclude cost from the vaccine attributes.
3) Policy implication: Cost data can guide policymakers in optimizing vaccination policies regarding the pricing or provision of free COVID-19 vaccines.^22^ Although the COVID-19 vaccines were free of charge in the previous vaccination program, this does not imply that they will be free in the future. Including cost allows policymakers to allocate resources efficiently and address any financial burden on public health insurance.

In summary, while cost may not match real-world scenarios, it remains a crucial attribute for assessing vaccine value, policy optimization, and overall cost-effectiveness. We have added the reason for considering cost in the detailed description of the DCE design in Appendix S2 (**Page 2, Paragraph 2**):

“In the final step, seven attributes related to the COVID-19 vaccine were included within the discrete choice experiment (DCE) design based on the previous studies^23^ and semi-structured qualitative interviews: total price, risk of rare but serious side-effects from the vaccine, duration of protection, degree of efficacy, vaccine administration, frequency of vaccination and vaccination origin. Levels for all attributes are also derived from the qualitative results. Despite the vaccines being distributed free of charge during the survey, we included price as an attribute. The main reason is due to its significance in gauging respondents' willingness to pay for the vaccine. Price remains a critical factor influencing vaccine acceptance, even when it’s not directly borne by individuals. In our preliminary literature review and qualitative online interviews, price is always a crucial factor that influences respondents’ choices. Therefore, including price as an attribute provides valuable insights for policymakers to optimize future vaccination policies.”

*2. Why there were 193 participants excluded from the analysis? Please specify what you mean by inconsistent data.*

Thank you very much for your suggestion. To improve the clarity of what we meant by data inconsistency, we have added the following exclusion criteria explicitly in the data collection section (**Page 11, Line 188-193**):

“In total, 12,193 adults agreed to take the online survey, among which 193 samples were excluded for not meeting the following requirements: 1) the participants must complete this survey between the time period of 360-1000 seconds, and 2) the age, marital status and education status should be consistent. This resulted in 12,000 total valid samples to be included in our final analysis.”

In **Appendix S2**, we also added the following quality control section:

“As a quality control measure, the participants must finish this survey between the time period of 360s and 1000s and verify understanding of the details before signing. In addition, we also checked the consistency of the survey data in age, marital status, and education status to exclude the participants with inconsistent responses.”

*3. Since the authors made use of an online survey platform in recruitment, it is usually more difficult to approach sufficient older participants using this method, particularly those aged above 70 years. Would the author also report the proportion of participants aged over 70 years and the population proportion as well? This is related to whether the sample is representative to the entire adult population or the working-age adult population.*

Thank you very much for your helpful insights regarding the age distribution of our study participants. We do not have participants aged over 70, but only 22.6% of participants aged 60-70.

While the online survey platform offers several advantages, including efficiency and broad reach, it presents difficulty in gaining access to an older population, especially those aged above 70 years. We acknowledge that our sample cannot fully represent the entire adult population due to this limitation. Our study primarily captures the perspectives of working-age adults (18-69 years specifically), and caution should be exercised when generalizing our results to older age groups. We have modified **Table 2** accordingly.

**Table 2. Sample descriptive characteristics (part)**

| **Characteristics** | **Participants, n (%)** | **Population of China, %** |
| --- | --- | --- |
| 18-39 years | 4,629 (38.6%) | 42.5% |
| 40-59 years | 4,659 (38.8%) | 43.9% |
| 60-69 years | 2712 (22.6%) | 14.6% |

We have also added the following statement in the limitations section (**Page 28, Line 526-529**):

“In particular, it presented challenges in reaching older individuals, and thus our sample may not fully represent the entire adult population, especially due to the absence of participants aged over 70.”

*4.* *In the DCE, was an exercise choice set used to help respondents get familiar with the task?*

Thank you very much for this insightful point. We agree with the reviewer that an exercise choice set would be helpful for respondents to get familiar with the task and improve the quality of the experiment data, and more frequently, in some studies, researchers often used dominant or repeated questions to exclude answers with bad quality.^24^ In the case of our DCE study, we intentionally included only the most critical questions in the DCE questions to keep the overall length of the experiment at approximately 10 minutes to minimize the time burden to the respondents. Moreover, we ran a pilot study prior to the formal DCE data collection to test respondents’ understanding of choice contexts^25^ and found that nearly all participants passed the internal consistency test and had no cognitive difficulties in understanding the choice set. These preliminary observations reassured us that having an additional exercise choice set may not be necessary (but could increase the time burden) in our study. For these reasons, we did not include the exercise choice set.

We have added the following explanation for not adding the exercise choice set (**Page 8-9, Line 159-164**):

“We did not add an exercise choice set to help respondents get familiar with the choice tasks due to the following reasons. First, nearly all participants passed the internal consistency test in the pilot study and had no cognitive difficulties in understanding the choice set. Second, it takes approximately 10 minutes to finish the actual choice set, and so an additional exercise choice set would increase the time burden.”

**References:**

1. World Health Organization. COVID-19 Vaccines with WHO Emergency Use Listing 2023 [Available from: <https://extranet.who.int/pqweb/vaccines/vaccinescovid-19-vaccine-eul-issued> accessed February 22, 2023.

2. Dong D, Xu RH, Wong EL-y, et al. Public preference for COVID-19 vaccines in China: A discrete choice experiment. *Health Expectations* 2020;23(6):1543-78. doi: <https://doi.org/10.1111/hex.13140>

3. Xinhua. China to offer free COVID-19 vaccination to citizens: official 2021 [accessed April 5 2024.

4. Azarpanah H, Farhadloo M, Vahidov R, et al. Vaccine hesitancy: evidence from an adverse events following immunization database, and the role of cognitive biases. *BMC Public Health* 2021;21:1-13.

5. Du Y, Jin C, Jit M, et al. Influenza vaccine uptake among children and older adults in China: a secondary analysis of a quasi-experimental study. *BMC Infect Dis* 2023;23(1):225.

6. Ye C, Zhu W, Yu J, et al. Low coverage rate and awareness of influenza vaccine among older people in Shanghai, China: A cross-sectional study. *Human Vaccines & Immunotherapeutics* 2018;14(11):2715-21.

7. Niu G, Zhao G. Living condition among China’s rural–urban migrants: Recent dynamics and the inland–coastal differential. *Housing Studies* 2018;33(3):476-93.

8. Crawshaw J, Konnyu K, Castillo G, et al. Behavioural determinants of COVID-19 vaccination acceptance among healthcare workers: A rapid review. *Public Health* 2022;210:123-33.

9. Xu B, Gao X, Zhang X, et al. Real-World Acceptance of COVID-19 Vaccines among Healthcare Workers in Perinatal Medicine in China. *Vaccines* 2021;9(7):704. doi: 10.3390/vaccines9070704

10. Peterson CJ, Lee B, Nugent K. COVID-19 vaccination hesitancy among healthcare workers—a review. *Vaccines* 2022;10(6):948.

11. Bauernfeind S, Hitzenbichler F, Huppertz G, et al. Brief report: attitudes towards Covid-19 vaccination among hospital employees in a tertiary care university hospital in Germany in December 2020. *Infection* 2021;49(6):1307-11.

12. Viswanath K, Bekalu M, Dhawan D, et al. Individual and social determinants of COVID-19 vaccine uptake. *BMC public health* 2021;21(1):818.

13. Shakeel CS, Mujeeb AA, Mirza MS, et al. Global COVID-19 vaccine acceptance: a systematic review of associated social and behavioral factors. *Vaccines* 2022;10(1):110.

14. Dhawan D, Bekalu M, Pinnamaneni R, et al. COVID-19 news and misinformation: do they matter for public health prevention? *Journal of health communication* 2021;26(11):799-808.

15. Zhao S, Hu S, Zhou X, et al. The prevalence, features, influencing factors, and solutions for COVID-19 vaccine misinformation: systematic review. *JMIR public health and surveillance* 2023;9(1):e40201.

16. Bekalu MA, Dhawan D, McCloud R, et al. Adherence to COVID-19 mitigation measures among American adults: the need for consistent and unified messaging. *Health Education Research* 2021;36(2):178-91.

17. Chen Y, Wang J, Yi M, et al. The COVID-19 vaccination decision-making preferences of elderly people: a discrete choice experiment. *Scientific Reports* 2023;13(1) doi: 10.1038/s41598-023-32471-1

18. Wang J, Lyu Y, Zhang H, et al. Willingness to pay and financing preferences for COVID-19 vaccination in China. *Vaccine* 2021;39(14):1968-76.

19. Zhou HJ, Pan L, Shi H, et al. Willingness to pay for and willingness to vaccinate with the COVID-19 vaccine booster dose in China. *Frontiers in pharmacology* 2022;13:1013485.

20. Pan S-J, Yang Y-P, Zhang M-X, et al. Willingness to pay for booster dose of COVID-19 vaccine among healthcare workers in Taizhou,China. *Human Vaccines &amp; Immunotherapeutics* 2022;18(5):1-5. doi: 10.1080/21645515.2022.2063629

21. Zhou Y, Zhang J, Wu W, et al. Willingness to receive future COVID-19 vaccines following the COVID-19 epidemic in Shanghai, China. *BMC Public Health* 2021;21(1):1103. doi: 10.1186/s12889-021-11174-0

22. Wong LP, Lee HY, Alias H, et al. Cost-based COVID-19 vaccination and willingness to pay: A post-pandemic review. *Human Vaccines &amp; Immunotherapeutics* 2024;20(1) doi: 10.1080/21645515.2024.2313860

23. Schwarzinger M, Watson V, Arwidson P, et al. COVID-19 vaccine hesitancy in a representative working-age population in France: a survey experiment based on vaccine characteristics. *The Lancet Public Health* 2021;6(4):e210-e21. doi: 10.1016/s2468-2667(21)00012-8

24. Abiiro GA, Torbica A, Kwalamasa K, et al. Eliciting community preferences for complementary micro health insurance: A discrete choice experiment in rural Malawi. *Social Science & Medicine* 2014;120:160-68. doi: <https://doi.org/10.1016/j.socscimed.2014.09.021>

25. Lancsar E, Louviere J. Conducting Discrete Choice Experiments to Inform Healthcare Decision Making. *PharmacoEconomics* 2008;26(8):661-77. doi: 10.2165/00019053-200826080-00004
